# Short-term risk and long-term incidence rate of infection and malignancy with IL-17 and IL-23 inhibitors in adult patients with psoriasis and psoriatic arthritis: a systematic review and meta-analysis

**DOI:** 10.3389/fimmu.2023.1294416

**Published:** 2023-11-29

**Authors:** Shuwei Wu, Yuanyuan Xu, Lihua Yang, Linghong Guo, Xian Jiang

**Affiliations:** ^1^ Department of Dermatology, West China Hospital, Sichuan University, Chengdu, China; ^2^ Laboratory of Dermatology, Clinical Institute of Inflammation and Immunology, Frontiers Science Center for Disease-related Molecular Network, West China Hospital, Sichuan University, Chengdu, China; ^3^ Tianfu Jincheng Laboratory & Institute of Future Medical Innovation, City of Future Medicine, Chengdu, China

**Keywords:** psoriasis, psoriatic arthritis, malignancy, infection, biologics, safety

## Abstract

The risk of infection and malignancy may be a concern for patients with psoriasis receiving interleukin (IL)-17 and IL-23 inhibitors, particularly with long-term treatments. We aimed to estimate the short-term risks and long-term incidence rates of infection and malignancy with IL-17 or IL-23 antagonists in adult patients with psoriasis and psoriatic arthritis through this comprehensive meta-analysis (PROSPERO registration number: CRD42022363127). We searched PubMed, MEDLINE, Web of Science and ClinicalTrials.gov until May 17, 2023 for randomized placebo-controlled trials and long-term (≥ 52 weeks) open-label extension studies. The estimates of short-term risk ratios (RRs) and long-term exposure-adjusted incidence rates (EAIRs) were pooled using R software 4.1.1 and STATA 16.0. This review included 45 randomized placebo-controlled studies and 27 open-label extension studies. Short-term RRs of serious infection, overall infection and malignancy were 1.45 (95% confidence intervals, 95% CI: 0.81-2.59), 1.20 (95% CI: 1.06-1.35), 0.83 (95% CI: 0.41-1.71) with IL-17 inhibitors; and 0.68 (95% CI: 0.38-1.22), 1.13 (95% CI: 1.00-1.28), 0.87 (95% CI: 0.37-2.04) with IL-23 inhibitors. Increased short-term risks of nasopharyngitis and *Candida* infection with IL-17 inhibitors were found. Long-term EAIRs of serious infection, overall infection, nonmelanoma skin cancer (NMSC), malignancies excluding NMSC, nasopharyngitis and upper respiratory tract infection were 1.11/100 patient-years (PYs), 57.78/100PYs, 0.47/100PYs, 0.24/100PYs, 15.07/100PYs, 8.52/100PYs, 3.41/100PYs with IL-17 inhibitors; and 1.09/100PYs, 48.50/100PYs, 0.40/100PYs, 0.43/100PYs, 10.75/100PYs, 5.84/100PYs with IL-23 inhibitors. Long-term EAIR of *Candida* infection was 3.41/100PYs with IL-17 inhibitors. No active or reactivated tuberculosis was ever reported in all the trials, and only a few cases of latent tuberculosis, hepatitis, and herpes zoster were reported during the long-term extension periods. No evidence of increased EAIRs of infection and malignancy with longer durations was found. Our study suggested that short-term risk and long-term incidence of infections and malignancies in psoriasis patients receiving IL-17 inhibitors and IL-23 inhibitors are generally low. However, close monitoring is required for nasopharyngitis and *Candida* infection with IL-17 inhibitors.

**Systematic Review Registration:**

https://www.crd.york.ac.uk/PROSPERO/, identifier CRD42022363127.

## 1 Introduction

Psoriasis is a chronic, inflammatory and immune-mediated skin disorder occurring worldwide, with plaque psoriasis (PsO) being the most common phenotype (1). Psoriatic arthritis (PsA), affecting approximately 30% of psoriasis patients, is a progressive and inflammatory musculoskeletal disorder resulting in declined quality of life, disability and early mortality (2, 3). PsO and PsA are associated with an increased prevalence of comorbidities, including cardiovascular disease, metabolic syndromes, and depression, which lead to substantial burdens for individual patients and society (4).

Currently, a variety of cytokines have been found to play pivotal roles in the pathogenesis of psoriasis, including interleukin (IL)-17 and IL-23 (5). A number of therapeutic agents targeting the IL-17 or IL-23 pathway have been approved in the treatment of PsO and PsA, and they have demonstrated excellent efficacy in both clinical trials and real-world settings (6). With these biologics, complete or nearly complete clearance of psoriatic lesions is now attainable. However, in addition to being implicated in the pathogenesis of psoriasis, the IL-17 family and IL-23 perform fundamental functions in innate and adaptive immunity (7, 8). Hence, concerns about infection and malignancy associated with deficiency of IL-17 or IL-23 immunity have been raised, especially in patients receiving long-term therapies (9).

To date, IL-17 inhibitors and IL-23 inhibitors have been proved generally safe and well-tolerated (6). However, individual trials lack large sample sizes or long-term trial durations to detect the risk of rare adverse events (AEs) such as serious infections, rare infections and malignancies (10). Furthermore, long-term safety is one of the key considerations in the treatment strategy for psoriasis, while most meta-analyses focused on the randomized controlled trials (RCTs), whose durations were routinely limited to tens of weeks (11). It is unclear, however, whether the short-term safety data could be generalized to long-term treatments. There is growing evidence from open-label extension studies demonstrating the long-term efficacy and safety profiles, with the follow-up periods extending up to several years (11). Nevertheless, to the best of our knowledge, comprehensive studies that generalize and quantify the current safety data, particularly the long-term data, are still lacking (11, 12).

To evaluate the short- and long-term safety profile of IL-17 or IL-23 inhibitors, we conducted this systematic review and meta-analysis, which assessed the short-term risk and long-term incidence rate of (1) overall infection, (2) serious infection, (3) malignancy, (4) nasopharyngitis, (5) upper respiratory tract infection, (6) *Candida* infection, (7) tuberculosis, (8) hepatitis, and (9) herpes zoster with IL-17 inhibitors and IL-23 inhibitors in adult patients with PsO or PsA.

## 2 Methods

### 2.1 Study design and systematic literature search

This study was designed as a systematic review and meta-analysis, according to the Preferred Reporting Item for Systematic Reviews and Meta-Analyses (PRISMA) guidelines (**Table S1**). A predefined protocol (PROSPERO registration number: CRD42022363127) was used to conduct the literature search, study selection, quality assessment, data extraction, and statistical analyses. The literature search was performed in PubMed, MEDLINE, Web of Science and ClinicalTrials.gov until May 17, 2023 for randomized placebo-controlled trials and long-term extension studies of IL-17 or IL-23 inhibitors approved for PsO and PsA. The search terms included “psoriasis or psoriatic arthritis”, combined with “IL-17 inhibitors or IL-23 inhibitors or IL-17 antagonists or IL-23 antagonists”. Publications were limited to the English language. Detailed searching strategy and results were summarized in the **Supplementary Materials**. Reference lists of selected articles were hand-searched to retrieve more literature.

### 2.2 Selection and quality assessment

We included (1) phase 2 or 3 randomized placebo-controlled trials; (2) open-label extension (OLE) studies with maintenance duration of more than 52 weeks. The included trials were required to report at least one of the primary outcomes: serious infection, overall infection or malignancy. Studies were excluded if they were not clinical trials on adult patients, or not reporting original data. Studies on IL-17/IL-23 inhibitors combined with other drugs instead of monotherapy were also excluded. The titles and abstracts of potentially eligible articles were screened by two independent investigators (S Wu and Y Xu). Afterwards, the full texts of the remaining studies were reviewed to identify if they met the inclusion criteria. Discrepancies between the investigators regarding the inclusion of the studies were resolved by discussion and consultation of a third researcher (L Guo). The risk of bias was evaluated by two independent reviewers (S Wu and Y Xu), using the Cochrane risk of bias tool for randomized studies of interventions (13). The divergence of opinions was resolved by consultation of another researcher (L Guo). The results were summarized and presented using Review Manager 5.4.1.

### 2.3 Outcomes, definitions and interventions

The primary outcomes of our study were the short-term risks and long-term incidence rates of (1) serious infection, (2) overall infection, (3) malignancy. We identified serious infections as infections considered to be serious adverse events (SAEs) by researchers of the trials. Malignancies were divided into nonmelanoma skin cancer (NMSC) and malignancies excluding NMSC in open-label extension studies according to the original literature. Secondary outcomes of our study were the short-term risks and long-time incidence rates of (1) nasopharyngitis, (2) upper respiratory tract infection, (3) *Candida* infection, (4) tuberculosis (latent or active), (5) hepatitis, (6) herpes zoster. The original literature researching IL-23 inhibitors lacked the data of *Candida* infection. Nasopharyngitis, upper respiratory tract infection, *Candida* infection, tuberculosis, hepatitis, and herpes zoster were selected as infections of special interest. Nasopharyngitis and upper respiratory tract infection are considered the most frequent AEs with IL-17 or IL-23 inhibitors, while *Candida* infection, tuberculosis, and herpes zoster are opportunistic infections which may be induced by deficiency of IL-17 or IL-23. Hepatitis here refers to infections with hepatitis virus, while other types of hepatitis not caused by infection, such as autoimmune hepatitis, were not included. IL-17 antagonists approved for the treatment of PsO or PsA include brodalumab, ixekizumab, secukinumab, and bimekizumab. IL-23 antagonists approved for the treatment of PsO or PsA include guselkumab, risankizumab, and tildrakizumab. Secukinumab and ixekizumab are monoclonal antibodies selectively binding IL-17A. Brodalumab targets the IL-17 receptor A unit (IL-17RA), inhibiting IL-17A, IL-17F, IL-17C and IL-17E/IL-25. Bimekizumab selectively neutralizes both IL-17A and IL-17F. Guselkumab, risankizumab and tildrakizumab are monoclonal antibodies blocking the p19 subunit of IL-23.

### 2.4 Data extraction and analyses

For included publications, the following information was collected: author, year of publication, trial name, ClinicalTrials.gov identifier, study design and phase, and type of disease. For RCTs, the following data were extracted: the number of patients in treatment and placebo groups, treatment dose, duration of the placebo-controlled periods, the number of serious infections, overall infections, malignancies, nasopharyngitis, upper respiratory tract infection and *Candida* infection, and the type of malignancies in both the treatment group and the placebo group. For OLE studies, we extracted the following data: treatment dose, number of patients, duration of the open-label extension periods, total patient-years (PYs) of exposure, exposure-adjusted incidence rate (EAIR) with 95% CIs of serious infection, overall infection, NMSC, malignancies excluding NMSC, nasopharyngitis, upper respiratory tract infection and *Candida* infection. Two reviewers (S Wu, L Yang) independently performed the data extraction. Disagreements were resolved by discussion and consultation of the third researcher (L Guo), and reviewers were unanimous in their final decisions. For AEs with limited data which were hard to be estimated by quantitative meta-analyses, qualitative descriptions, evaluations and summary tables were presented.

The numbers of subjects with infection or malignancy in the treatment and placebo groups from the eligible RCTs were recorded. Patients who received any dose of the biologics were included in the outcome measures. Risk ratios (RRs) based on the number of patients with infections or malignancies and the number of patients in each group were calculated. A pooled estimate of the RR with associated 95% confidence intervals (CIs) was produced for each outcome, with each result presented with forest plots. Heterogeneity testing was performed using the I^2^ test and Q-test. A common-effects model and a random-effects model were both calculated. A random-effects model would be adapted if I^2^ ≥50%. Subgroup analyses were conducted by biologic and indication. We performed funnel plots and “peters” tests (more than 10 studies) to estimate the publication bias (14). Sensitivity analyses were performed to measure the consistency and robustness of the outcomes. Statistical analyses were performed by R software 4.1.1 and the “meta” package. P-values less than 0.05 were considered significant.

The long-term incidence rates of infection and malignancy were determined by EAIRs with 95% CIs, calculated by events/100PYs. Data of 95% CIs were calculated based on Poisson distribution if they were not provided by the original literature (15). Data with durations of more than 52 weeks were included and analyzed. Patients who received any doses of the biologics were included in the final measures. We produced a pooled estimate of the EAIR of each outcome, with the results presented with forest plots. The results were stratified by years, aimed to determine EAIRs of different treatment durations. Statistical analyses were performed using STATA 16.0. P-values less than 0.05 were considered significant.

## 3 Results

### 3.1 Search results and trial characteristics

**Figure 1:**
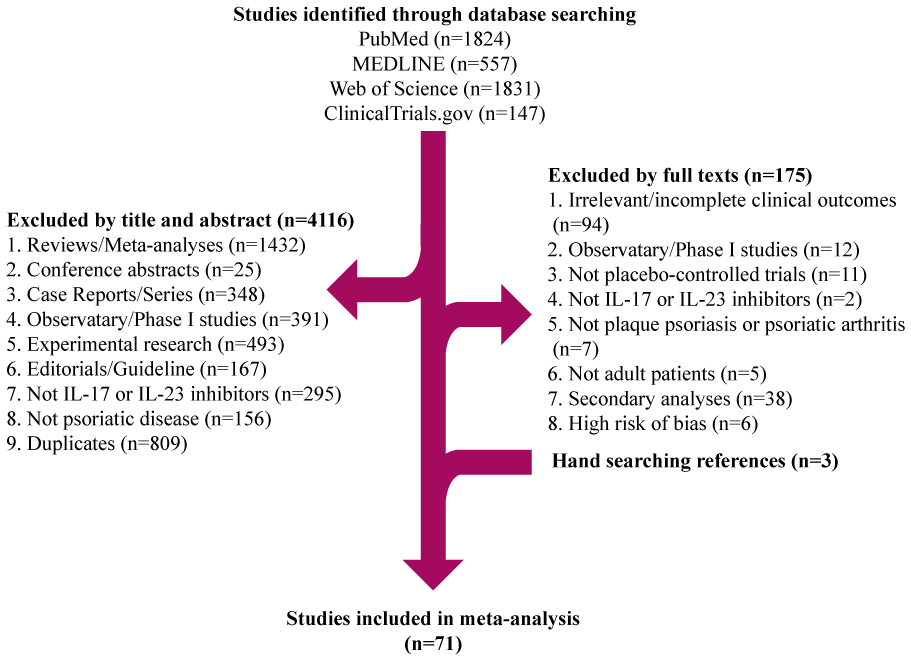
Flow diagram of selection of literature.

A total of 4,359 potentially relevant publications were identified through database searching, of which 4,116 were excluded after screening the title or abstract, 174 were excluded after full-text review. The researchers agreed on the major part of the study inclusion and data extraction, except for 6 publications, which were solved by consultation of the third researcher (L Guo). Then three publications were included, and three were excluded (1 study on children; 1 study on psoriasis erythrodermic; 1 study not placebo-controlled). Ultimately, 71 publications were qualified for inclusion (**Figure 1**) (16–84). A total of 25,496 patients were included in 44 randomized placebo-controlled studies (16–59) and 17,591 patients with 42,043.3 PYs of total exposure were included in 28 OLE studies (17, 31, 48, 60–84). The characteristics of RCTs were summarized in **Table 1**, and the characteristics of OLE studies were summarized in **Table 2**. All the included studies researched monotherapy of IL-17 or IL-23 inhibitors, with no other drug combined throughout all the trial periods. The reported cases of tuberculosis, hepatitis, and herpes zoster were summarized in **Tables 3** and **4**, as some of these AEs were so rare that meta-analyses were not able to be performed. Original data extracted from the included literatures were summarized in **Tables S2**-**S4**. Even when the risk of blinding was unclear in some trials, the overall risk of bias was low. The detailed risk of bias of each included study assessed by the Cochrane risk of bias tool for randomized studies of interventions was presented in **Figure S1**.

**Table 1:** Trial characteristics of randomized placebo-controlled trials.

Source	Trial Name/ClinicalTrials.gov identifier	Study Design/Phase	Disease	Placebo-Controlled Period (weeks)	Treatment (dose)	Number of Patients	
						Treatment Group	Placebo Group
Papp et al., 2016 (16)	AMAGINE-1/NCT01708590	RCT^1^/ Phase 3	PsO^2^	12	Brodalumab (140 mg, Q2W^4^) Brodalumab (210 mg, Q2W)	219 222	220
Lebwohl et al., 2015 (17)	AMAGINE-2/NCT01708603	RCT/ Phase 3	PsO	12	Brodalumab (140 mg, Q2W) Brodalumab (210 mg, Q2W)	610 612	309
Lebwohl et al., 2015 (17)	AMAGINE-3/NCT01708629	RCT/ Phase 3	PsO	12	Brodalumab (140 mg, Q2W) Brodalumab (210 mg, Q2W)	629 624	315
Mease et al., 2021 (18)	AMVISION-1/NCT02029495; AMVISION-2/NCT02024646	RCT/ Phase 3	PsA^3^	16	Brodalumab (140 mg, at weeks 0, 1, then Q2W) Brodalumab (210 mg, at weeks 0, 1, then Q2W)	318 321	320
Seo et al., 2021 *(* 19)	NCT02982005	RCT/ Phase 3	PsO	12	Brodalumab (210 mg, at weeks 0, 1, then Q2W)	40	22
Mease et al., 2014 (20)	NCT01516957	RCT/ Phase 2	PsA	12	Brodalumab (140 mg, at weeks 0, 1, then Q2W) Brodalumab (280 mg, at weeks 0, 1, then Q2W)	56 56	55
Papp et al., 2012 (21)	NCT00975637	RCT/ Phase 2	PsO	12	Brodalumab (70 mg, at weeks 0, 1, then Q2W) Brodalumab (140 mg, at weeks 0, 1, then Q2W) Brodalumab (210 mg, at weeks 0, 1, then Q2W) Brodalumab (280 mg, monthly)	39 39 40 41	38
Nakagawa et al., 2016 (22)	NCT01748539	RCT/ Phase 2	PsO	12	Brodalumab (70 mg, at weeks 0, 1, then Q2W) Brodalumab (140 mg, at weeks 0, 1, then Q2W) Brodalumab (210 mg, at weeks 0, 1, then Q2W)	39 37 37	38
Gordon et al., 2016 (23)	UNCOVER-1/NCT01474512; UNCOVER-2/NCT01597245; UNCOVER-3/ NCT01646177	RCT/ Phase 3	PsO	12	Ixekizumab (160 mg at week 0, then 80 mg Q2W) Ixekizumab (160 mg at week 0, then 80 mg Q4W^5^)	1167 1161	791
Mease et al., 2017 (24)	SPIRIT-P1/NCT01695239	RCT/ Phase 3	PsA	24	Ixekizumab (160 mg at week 0, then 80 mg Q2W) Ixekizumab (160 mg at week 0, then 80 mg Q4W)	102 107	106
Nash et al., 2017 (25)	SPIRIT-P2/NCT02349295	RCT/ Phase 3	PsA	24	Ixekizumab (160 mg at week 0, then 80 mg Q2W) Ixekizumab (160 mg at week 0, then 80 mg Q4W)	123 122	118
Ryan et al., 2018 (26)	IXORA-Q/NCT02718898	RCT/ Phase 3	PsO	12	Ixekizumab (160 mg at week 0, then 80 mg Q2W)	75	74
Leonardi et al., 2012 (27)	NCT01107457	RCT/ Phase 2	PsO	20	Ixekizumab (10 mg, at weeks 0, 2, 4, 8, 12, and 16) Ixekizumab (25 mg, at weeks 0, 2, 4, 8, 12, and 16) Ixekizumab (75 mg, at weeks 0, 2, 4, 8, 12, and 16) Ixekizumab (150 mg, at weeks 0, 2, 4, 8, 12, and 16)	28 30 29 28	27
Langley et al., 2014 (28)	ERASURE/NCT01365455	RCT/ Phase 3	PsO	12	Secukinumab (150 mg, QW^6^ for 5 weeks, then Q4W) Secukinumab (300 mg, QW for 5 weeks, then Q4W)	245 245	247
Langley et al., 2014 (28)	FIXTURE/NCT01358578	RCT/ Phase 3	PsO	12	Secukinumab (150 mg, QW for 5 weeks, then Q4W) Secukinumab (300 mg, QW for 5 weeks, then Q4W)	327 326	327
Mease et al., 2015 (29)	FUTURE1/NCT01392326	RCT/ Phase 3	PsA	16	Secukinumab (10 mg per kilogram at weeks 0, 2, and 4, then 75 mg Q4W) Secukinumab (10 mg per kilogram at weeks 0, 2, and 4, then 150 mg Q4W)	202 202	202
McInnes et al., 2015 (30)	FUTURE2/NCT01752634	RCT/ Phase 3	PsA	16	Secukinumab (75 mg, QW for 4 weeks, then Q4W) Secukinumab (150 mg, QW for 4 weeks, then Q4W) Secukinumab (300 mg, QW for 4 weeks, then Q4W)	99 100 100	98
Nash et al., 2018 (31)	FUTURE3/NCT01989468	RCT/ Phase 3	PsA	16	Secukinumab (150 mg, QW for 4 weeks, then Q4W) Secukinumab (300 mg, QW for 4 weeks, then Q4W)	138 139	137
Mease et al., 2018 (32)	FUTURE5/NCT02404350	RCT/ Phase 3	PsA	24	Secukinumab (150 mg with loading dose, QW for 4 weeks, then Q4W) Secukinumab (150 mg without loading dose, QW for 4 weeks, then Q4W) Secukinumab (300 mg, QW for 4 weeks, then Q4W)	822	332
Baraliakos et al., 2021 (33)	MAXIMISE/NCT02721966	RCT/ Phase 3	PsA	12	Secukinumab (150 mg, QW for 4 weeks, then Q4W) Secukinumab (300 mg, QW for 4 weeks, then Q4W)	165 167	166
Gottlieb et al., 2017 (34)	GESTURE/NCT01806597	RCT/ Phase 3	PsO	16	Secukinumab (150 mg, QW for 4 weeks, then Q4W) Secukinumab (300 mg, QW for 4 weeks, then Q4W)	68 69	68
Paul et al., 2015 (35)	JUNCTURE/ NCT01636687	RCT/ Phase 3	PsO	12	Secukinumab (150 mg, QW for 4 weeks, then Q4W) Secukinumab (300 mg, QW for 4 weeks, then Q4W)	61 60	61
Bagel et al., 2017 (36)	NCT02267135	RCT/ Phase 3	PsO	24	Secukinumab (300 mg, QW for 4 weeks, then Q4W)	50	51
McInnes et al., 2014 (37)	NCT00809614	RCT/ Phase 2	PsA	24	Secukinumab (10mg/kg, on day 1 and day 22)	28	14
Nguyen et al., 2022 (38)	CHOICE/ NCT02798211	RCT/ Phase 2	PsO, PsA	16	Secukinumab (150 mg, QW for 4 weeks, then Q4W) Secukinumab (300 mg, QW for 4 weeks, then Q4W)	103 103	52
Papp et al., 2013 (39)	NCT01071252	RCT/ Phase 2	PsO	12	Secukinumab (1*25 mg, at weeks 0, 4 and 8) Secukinumab (3*25 mg, at weeks 0, 4 and 8) Secukinumab (3*75 mg, at weeks 0, 4 and 8) Secukinumab (3*150 mg, at weeks 0, 4 and 8)	29 26 21 27	22
Rich et al., 2013 (40)	NCT00941031	RCT/ Phase 2	PsO	12	Secukinumab (150 mg, at week 0) Secukinumab (150 mg, at weeks 0, 4 and 8) Secukinumab (150 mg, at weeks 0, 1, 2 and 4)	66 138 133	67
Gordon et al., 2021 (41)	BE READY/ NCT03410992	RCT/ Phase 3	PsO	16	Bimekizumab (320 mg Q4W)	349	86
McInne et al., 2023 (42)	BE OPTIMAL/NCT03895203	RCT/ Phase 3	PsA	16	Bimekizumab (160 mg Q4W)	431	281
Merola et al., 2023 (43)	BE COMPLETE/ NCT03896581	RCT/ Phase 3	PsA	16	Bimekizumab (160 mg Q4W)	267	132
Reich et al., 2021 (44)	BE VIVID/ NCT03370133	RCT/ Phase 3	PsO	16	Bimekizumab (320 mg Q4W)	321	83
Ritchlin et al., 2020 (45)	BE ACTIVE/NCT02969525	RCT/ Phase 2	PsA	12	Bimekizumab (160 mg Q4W) Bimekizumab (160 mg Q4W) Bimekizumab (160 mg Q4W, loading dose) Bimekizumab (320 mg Q4W)	39 43 41 41	42
Blauvelt et al., 2017 (46)	VOYAGE 1/NCT02207231	RCT/ Phase 3	PsO	16	Guselkumab (100 mg, at weeks 0 and 4, then Q8W^7^)	329	174
Reich et al., 2017 (47)	VOYAGE 2/NCT02207244	RCT/ Phase 3	PsO	16	Guselkumab (100 mg, at weeks 0 and 4, then Q8W)	494	248
Coates et al., 2022 (48)	COSMOS/ NCT03796858	RCT/ Phase 3	PsA	24	Guselkumab (100 mg, at weeks 0 and 4, then Q8W)	189	96
Deodhar et al., 2020 (49)	DISCOVER-1/ NCT03162796	RCT/ Phase 3	PsA	24	Guselkumab (100 mg, Q4W) Guselkumab (100 mg, at weeks 0 and 4, then Q8W)	128 127	126
Mease et al., 2020 (50)	DISCOVER-2/ NCT03158285	RCT/ Phase 3	PsA	24	Guselkumab (100 mg, Q4W) Guselkumab (100 mg, at weeks 0 and 4, then Q8W)	245 248	246
Ohtsuki et al., 2018 (51)	NCT02325219	RCT/ Phase 3	PsO	16	Guselkumab (50 mg, at weeks 0 and 4, then Q8W) Guselkumab (100 mg, at weeks 0 and 4, then Q8W)	65 63	64
Deodhar et al., 2018 (52)	NCT02319759	RCT/ Phase 2	PsA	24	Guselkumab (100 mg, at weeks 0 and 4, then Q8W)	100	49
Gordon et al., 2015 (53)	NCT01483599	RCT/ Phase 2	PsO	16	Guselkumab (5 mg, at weeks 0 and 4, then Q12W^8^) Guselkumab (15 mg Q8W) Guselkumab (50 mg, at weeks 0 and 4, then Q12W) Guselkumab (100 mg Q8W) Guselkumab (200 mg, at weeks 0 and 4, then Q12W)	41 41 42 42 41	42
Gordon et al., 2018 (54)	UltIMMa-1/ NCT02684370	RCT/ Phase 3	PsO	16	Risankizumab (150 mg, at weeks 0, 4, and 16)	304	102
Gordon et al., 2018 (54)	UltIMMa-2/ NCT02684357	RCT/ Phase 3	PsO	16	Risankizumab (150 mg, at weeks 0, 4, and 16)	294	98
Kristensen et al., 2022 (55)	KEEPsAKE 1/ NCT03675308	RCT/ Phase 3	PsA	24	Risankizumab (150 mg, at weeks 0, 4, and 16)	483	481
Östör et al., 2022 (56)	KEEPsAKE 2/ NCT03671148	RCT/ Phase 3	PsA	24	Risankizumab (150 mg, at weeks 0, 4, and 16)	224	219
Blauvelt et al., 2020 (57)	NCT02672852	RCT/ Phase 3	PsO	16	Risankizumab (150 mg, at weeks 0, 4, and 16)	407	100
Reich et al., 2017 (58)	reSURFACE 1/ NCT01722331	RCT/ Phase 3	PsO	16	Tildrakizumab (100 mg, at weeks 0, 4, and 16) Tildrakizumab (200 mg, at weeks 0, 4, and 16)	309 308	154
Reich et al., 2017 (58)	reSURFACE 2/ NCT01729754	RCT/ Phase 3	PsO	16	Tildrakizumab (100 mg, at weeks 0, 4, and 16) Tildrakizumab (200 mg, at weeks 0, 4, and 16)	307 314	156
Papp et al., 2015 (59)	NCT01225731	RCT/ Phase 2	PsO	16	Tildrakizumab (5 mg, at weeks 0, 4, and 16) Tildrakizumab (25 mg, at weeks 0, 4, and 16) Tildrakizumab (100 mg, at weeks 0, 4, and 16) Tildrakizumab (200 mg, at weeks 0, 4, and 16)	42 91 89 86	45

^1^RCT, randomized controlled trial; ^2^PsO, psoriasis; ^3^PsA, psoriatic arthritis; ^4^Q2W, every 2 weeks; ^5^Q4W, every 4 weeks; ^6^QW, every week; ^7^Q8W, every 8 weeks; ^8^Q12W, every 12 weeks.

**Table 2:** Trial characteristics of open-label extension studies.

Source	Trial Name/ClinicalTrials.gov identifier	Study Design/Phase	Disease	Treatment (dose)	Number of Patients	Duration of Open-label Extension Period	Total Patient-years of Exposure
Papp et al., 2020 (60)	AMAGINE-1/NCT01708590	OLE^1^/ Phase 3	PsO^2^	Brodalumab (140mg or 210 mg, Q2W^4^)	648	1 year 120 weeks	594.6 1338.8
Lebwohl et al., 2015 (17)	AMAGINE-2/NCT01708603	OLE/ Phase 3	PsO	Brodalumab (140mg or 210 mg, Q2W)	1567	52 weeks	1366.8
Lebwohl et al., 2015 (17)	AMAGINE-3/NCT01708629	OLE/ Phase 3	PsO	Brodalumab (140mg or 210 mg, Q2W)	1613	52 weeks	1410.8
Reich et al., 2022 (61)	AMAGINE-2/NCT01708603; AMAGINE-3/NCT01708629	OLE/ Phase 3	PsO	Brodalumab (140mg, Q2W) Brodalumab (210 mg, Q2W)	339 595	120 weeks	646 1152.2
Lebwohl et al., 2019 (62)	NCT01101100	OLE/ Phase 2	PsO	Brodalumab (210 mg, Q2W)	181	1 year 2 years 3 years 4 years 5 years 6 years	172.9 330.1 475.2 611 725.7 731.7
Blauvelt et al., 2021 (63)	UNCOVER-3/NCT01646177	OLE/ Phase 3	PsO	Ixekizumab (160 mg at week 0, then 80 mg Q2W or Q4W^5^)	335 385	1 year 5 years	355.5 1493.8
van der Heijde et al., 2018 (64)	SPIRIT-P1/NCT01695239	OLE/ Phase 3	PsA^3^)	Ixekizumab (160 mg at week 0, then 80 mg Q2W) Ixekizumab (160 mg at week 0, then 80 mg Q4W)	189 197	52 weeks	133 134.7
Chandran et al., 2020 (65)	SPIRIT-P1/NCT01695239	OLE/ Phase 3	PsA	Ixekizumab (160 mg at week 0, then 80 mg Q2W) Ixekizumab (160 mg at week 0, then 80 mg Q4W)	189 197	3 years	440 449
Genovese et al., 2018 (66)	SPIRIT-P2/NCT02349295	OLE/ Phase 3	PsA	Ixekizumab (160 mg at week 0, then 80 mg Q2W) Ixekizumab (160 mg at week 0, then 80 mg Q4W)	107 111	52 weeks	77.8 87.7
Gordon et al., 2014 (67)	NCT01107457	OLE/ Phase 2	PsO	Ixekizumab (6 doses at 0, 2, 4, 8, 12, and 16 weeks, then 120 mg Q4W)	120	52 weeks	170.1
Lacour et al., 2017 (68)	JUNCTURE/NCT01636687	OLE/ Phase 3	PsO	Secukinumab (150 mg or 300 mg, QW^6^ for 4 weeks, then Q4W)	177	52 weeks	159.7
Blauvelt et al., 2017 (69)	CLEAR/ NCT02074982	OLE/ Phase 3	PsO	Secukinumab (300 mg, QW for 4 weeks, then Q4W)	335	52 weeks	324.9
McInnes et al., 2017 (70)	FUTURE2/NCT01752634	OLE/ Phase 3	PsA	Secukinumab (75, 150 or 300 mg, QW for 4 weeks, then Q4W)	387	104 weeks	751.3
Nash et al., 2018 (31)	FUTURE3/NCT01989468	OLE/ Phase 3	PsA	Secukinumab (150 or 300 mg, QW for 4 weeks, then Q4W)	406	52 weeks	398.1
van der Heijde et al., 2020 (71)	FUTURE5/NCT02404350	OLE/ Phase 3	PsA	Secukinumab (150 mg, QW for 4 weeks, then Q4W) Secukinumab (300 mg, QW for 4 weeks, then Q4W)	593 371	52 weeks	512.2 303.2
Mease et al., 2021 (72)	FUTURE5/NCT02404350	OLE/ Phase 3	PsA	Secukinumab (150 or 300 mg, QW for 4 weeks, then Q4W)	964	2 years	1761.5
Bissonnette et al., 2018 (73)	SCULPTURE/NCT01640951	OLE/ Phase 2/3	PsO	Secukinumab (300mg, QW for 4 weeks, then Q4W, fixed interval)	168 168 168 142 134	1 year 2 years 3 years 4 years 5 years	168 330.8 479.6 616.1 758.1
Bissonnette et al., 2017 (74)	SCULPTURE/NCT01640951	OLE/ Phase 2/3	PsO	Secukinumab (300mg, QW for 4 weeks, then Q4W, retreated as needed)	172	3 years	470.6
Coates et al., 2022 (75)	BE ACTIVE/ NCT03347110	OLE/ Phase 2	PsA	Bimekizumab (160 mg Q4W); Bimekizumab (160 mg Q8W^7^)	206	152 weeks	570.1
Strober et al., 2023 (76)	BE RADIANT/NCT03536884	OLE/ Phase 3	PsO	Bimekizumab (320 mg Q4W)	373 336	1 year 2 years	373 672
Reich et al., 2020 (77)	VOYAGE 1/NCT02207231; VOYAGE 2/NCT02207244	OLE/ Phase 3	PsO	Guselkumab (100 mg, at weeks 0 and 4, then Q8W)	1221	100 weeks 156 weeks	2084 3222
Blauvelt et al., 2022 (78)	VOYAGE 1/NCT02207231; VOYAGE 2/NCT02207244	OLE/ Phase 3	PsO	Guselkumab (100 mg, at weeks 0 and 4, then Q8W)	1221	5 years	5254
Coates et al., 2022 (48)	COSMOS/ NCT03796858	OLE/ Phase 3	PsA	Guselkumab (100 mg, at weeks 0 and 4, then Q8W)	279	56 weeks	255.4
McInnes et al., 2021 (79)	DISCOVER-2/ NCT03158285	OLE/ Phase 3	PsA	Guselkumab (100 mg, Q4W) Guselkumab (100 mg, at weeks 0 and 4, then Q8W)	245 248	52 weeks	239 241
McInnes et al., 2022 (80)	DISCOVER-2/ NCT03158285	OLE/ Phase 3	PsA	Guselkumab (100 mg, Q4W) Guselkumab (100 mg, at weeks 0 and 4, then Q8W)	245 248	112 weeks	499 509
Gooderham et al., 2022 (81)	UltIMMa-1/NCT02684370; UltIMMa-2/NCT02684357	OLE/ Phase 3	PsO	Risankizumab (150 mg, at weeks 0 and 4, then Q12W^8^)	598	52 weeks	618
Gooderham et al., 2022 (81)	LIMMitless/NCT03047395	OLE/ Phase 3	PsO	Risankizumab (150 mg, at weeks 0 and 4, then Q12W)	525	172 weeks	1909.5
Papp et al., 2021 (82)	LIMMitless/NCT03047395	OLE/ Phase 3	PsO	Risankizumab (150 mg, at weeks 0 and 4, then Q12W)	897	208 weeks	3106.2
Reich et al., 2020 (83)	reSURFACE 1/ NCT01722331; reSURFACE 2/ NCT01729754	OLE/ Phase 3	PsO	Tildrakizumab (100 mg, at weeks 0 and 4, then Q12W) Tildrakizumab (200 mg, at weeks 0 and 4, then Q12W)	616 622	148 weeks	2014·49 2046·71
Thaci et al., 2021 (84)	reSURFACE 1/ NCT01722331; reSURFACE 2/ NCT01729754	OLE/ Phase 3	PsO	Tildrakizumab (100 mg, at weeks 0 and 4, then Q12W) Tildrakizumab (200 mg, at weeks 0 and 4, then Q12W)	872 928	256 weeks	2688.4 2753.5

^1^OLE, open-label extension; ^2^PsO, psoriasis; ^3^PsA, psoriatic arthritis; ^4^Q2W, every 2 weeks; ^5^Q4W, every 4 weeks; ^6^QW, every week; ^7^Q8W, every 8 weeks; ^8^Q12W, every 12 weeks.

**Table 3:** The number of tuberculosis, hepatitis, and herpes zoster in both treatment groups and placebo groups in the included RCTs.

Source	Trial Name/ClinicalTrials.gov identifier	Treatment (dose)	Treatment Group		Placebo Group	
			Number of patients	Number of events	Number of patients	Number of events
Tuberculosis						
Mease et al., 2014 (20)	NCT01516957	Brodalumab (140 mg, at weeks 0, 1, then Q2W) Brodalumab (280 mg, at weeks 0, 1, then Q2W)	56 56	0 0	55	0
Mease et al., 2017 (24)	SPIRIT-P1/NCT01695239	Ixekizumab (160 mg at week 0, then 80 mg Q2W) Ixekizumab (160 mg at week 0, then 80 mg Q4W)	102 107	0 0	106	0
Nash et al., 2017 (25)	SPIRIT-P2/NCT02349295	Ixekizumab (160 mg at week 0, then 80 mg Q2W) Ixekizumab (160 mg at week 0, then 80 mg Q4W)	123 122	0 0	118	0
Mease et al., 2015 (29)	FUTURE1/NCT01392326	Secukinumab (10 mg per kilogram at weeks 0, 2, and 4, then 75 mg Q4W) Secukinumab (10 mg per kilogram at weeks 0, 2, and 4, then 150 mg Q4W)	202 202	0 0	202	0
McInnes et al., 2015 (30)	FUTURE2/NCT01752634	Secukinumab (75 mg, QW for 4 weeks, then Q4W) Secukinumab (150 mg, QW for 4 weeks, then Q4W) Secukinumab (300 mg, QW for 4 weeks, then Q4W)	99 100 100	0 0 0	98	0
Mease et al., 2018 (32)	FUTURE5/NCT02404350	Secukinumab (150 mg with loading dose, QW for 4 weeks, then Q4W) Secukinumab (150 mg without loading dose, QW for 4 weeks, then Q4W) Secukinumab (300 mg, QW for 4 weeks, then Q4W)	822	0	332	0
Nguyen et al., 2022 (38)	CHOICE/ NCT02798211	Secukinumab (150 mg, QW for 4 weeks, then Q4W) Secukinumab (300 mg, QW for 4 weeks, then Q4W)	103 103	0 0	52	0
Gordon et al., 2021 (41)	BE READY/ NCT03410992	Bimekizumab (320 mg Q4W)	349	0	86	0
McInne et al., 2023 (42)	BE OPTIMAL/NCT03895203	Bimekizumab (160 mg Q4W)	431	0	281	0
Merola et al., 2023 (43)	BE COMPLETE/ NCT03896581	Bimekizumab (160 mg Q4W)	267	0	132	0
Reich et al., 2021 (44)	BE VIVID/ NCT03370133	Bimekizumab (320 mg Q4W)	321	0	83	0
Ritchlin et al., 2020 (45)	BE ACTIVE/NCT02969525	Bimekizumab (160 mg Q4W) Bimekizumab (160 mg Q4W) Bimekizumab (160 mg Q4W, loading dose) Bimekizumab (320 mg Q4W)	39 43 41 41	0 0 0 0	42	0
Reich et al., 2017 (47)	VOYAGE 2/NCT02207244	Guselkumab (100 mg, at weeks 0 and 4, then Q8W)	494	0	248	0
Deodhar et al., 2020 (49)	DISCOVER-1/ NCT03162796	Guselkumab (100 mg, Q4W) Guselkumab (100 mg, at weeks 0 and 4, then Q8W)	128 127	0 0	126	0
Mease et al., 2020 (50)	DISCOVER-2/ NCT03158285	Guselkumab (100 mg, Q4W) Guselkumab (100 mg, at weeks 0 and 4, then Q8W)	245 248	0 0	246	0
Ohtsuki et al., 2018 (51)	NCT02325219	Guselkumab (50 mg, at weeks 0 and 4, then Q8W) Guselkumab (100 mg, at weeks 0 and 4, then Q8W)	65 63	0 0	64	0
Deodhar et al., 2018 (52)	NCT02319759	Guselkumab (100 mg, at weeks 0 and 4, then Q8W)	100	0	49	0
Gordon et al., 2015 (53)	NCT01483599	Guselkumab (5 mg, at weeks 0 and 4, then Q12W^8^) Guselkumab (15 mg Q8W) Guselkumab (50 mg, at weeks 0 and 4, then Q12W) Guselkumab (100 mg Q8W) Guselkumab (200 mg, at weeks 0 and 4, then Q12W)	41 41 42 42 41	0 0 0 0 0	42	0
Gordon et al., 2018 (54)	UltIMMa-1/ NCT02684370	Risankizumab (150 mg, at weeks 0, 4, and 16)	304	0	102	0
Gordon et al., 2018 (54)	UltIMMa-2/ NCT02684357	Risankizumab (150 mg, at weeks 0, 4, and 16)	294	0	98	0
Kristensen et al., 2022 (55)	KEEPsAKE 1/ NCT03675308	Risankizumab (150 mg, at weeks 0, 4, and 16)	483	0	481	0
Östör et al., 2022 (56)	KEEPsAKE 2/ NCT03671148	Risankizumab (150 mg, at weeks 0, 4, and 16)	224	0	219	0
Blauvelt et al., 2020 (57)	NCT02672852	Risankizumab (150 mg, at weeks 0, 4, and 16)	407	0	100	0
Reich et al., 2017 (58)	reSURFACE 1/ NCT01722331	Tildrakizumab (100 mg, at weeks 0, 4, and 16) Tildrakizumab (200 mg, at weeks 0, 4, and 16)	309 308	0 0	154	0
Reich et al., 2017 (58)	reSURFACE 2/ NCT01729754	Tildrakizumab (100 mg, at weeks 0, 4, and 16) Tildrakizumab (200 mg, at weeks 0, 4, and 16)	307 314	0 0	156	0
Papp et al., 2015 (59)	NCT01225731	Tildrakizumab (5 mg, at weeks 0, 4, and 16) Tildrakizumab (25 mg, at weeks 0, 4, and 16) Tildrakizumab (100 mg, at weeks 0, 4, and 16) Tildrakizumab (200 mg, at weeks 0, 4, and 16)	42 91 89 86	0 0 0 0	45	0
Hepatitis						
Langley et al., 2014 (28)	FIXTURE/NCT01358578	Secukinumab (150 mg, QW for 5 weeks, then Q4W) Secukinumab (300 mg, QW for 5 weeks, then Q4W)	327 326	0 0	327	1
McInnes et al., 2015 (30)	FUTURE2/NCT01752634	Secukinumab (75 mg, QW for 4 weeks, then Q4W) Secukinumab (150 mg, QW for 4 weeks, then Q4W) Secukinumab (300 mg, QW for 4 weeks, then Q4W)	99 100 100	0 0 1	98	0
Mease et al., 2020 (50)	DISCOVER-2/ NCT03158285	Guselkumab (100 mg, Q4W) Guselkumab (100 mg, at weeks 0 and 4, then Q8W)	245 248	1 0	246	0
Ohtsuki et al., 2018 (51)	NCT02325219	Guselkumab (50 mg, at weeks 0 and 4, then Q8W) Guselkumab (100 mg, at weeks 0 and 4, then Q8W)	65 63	0 0	64	0
Herpes zoster						
Mease et al., 2021 (18)	AMVISION-1/NCT02029495; AMVISION-2/NCT02024646	Brodalumab (140 mg, at weeks 0, 1, then Q2W) Brodalumab (210 mg, at weeks 0, 1, then Q2W)	318 321	0 0	320	3
Mease et al., 2014 (20)	NCT01516957	Brodalumab (140 mg, at weeks 0, 1, then Q2W) Brodalumab (280 mg, at weeks 0, 1, then Q2W)	56 56	0 0	55	0
Papp et al., 2012 (21)	NCT00975637	Brodalumab (70 mg, at weeks 0, 1, then Q2W) Brodalumab (140 mg, at weeks 0, 1, then Q2W) Brodalumab (210 mg, at weeks 0, 1, then Q2W) Brodalumab (280 mg, monthly)	39 39 40 41	0 1 0 0	38	0
Gordon et al., 2016 (23)	UNCOVER-1/NCT01474512; UNCOVER-2/NCT01597245; UNCOVER-3/ NCT01646177	Ixekizumab (160 mg at week 0, then 80 mg Q2W) Ixekizumab (160 mg at week 0, then 80 mg Q4W)	1167 1161	0 0	791	0
Mease et al., 2017 (24)	SPIRIT-P1/NCT01695239	Ixekizumab (160 mg at week 0, then 80 mg Q2W) Ixekizumab (160 mg at week 0, then 80 mg Q4W)	102 107	1 0	106	0
Mease et al., 2020 (50)	DISCOVER-2/ NCT03158285	Guselkumab (100 mg, Q4W) Guselkumab (100 mg, at weeks 0 and 4, then Q8W)	245 248	0 0	246	0
Ohtsuki et al., 2018 (51)	NCT02325219	Guselkumab (50 mg, at weeks 0 and 4, then Q8W) Guselkumab (100 mg, at weeks 0 and 4, then Q8W)	65 63	0 0	64	0
Gordon et al., 2018 (54)	UltIMMa-2/ NCT02684357	Risankizumab (150 mg, at weeks 0, 4, and 16)	294	1	98	0
Kristensen et al., 2022 (55)	KEEPsAKE 1/ NCT03675308	Risankizumab (150 mg, at weeks 0, 4, and 16)	483	2	481	1
Östör et al., 2022 (56)	KEEPsAKE 2/ NCT03671148	Risankizumab (150 mg, at weeks 0, 4, and 16)	224	0	219	1
Blauvelt et al., 2020 (57)	NCT02672852	Risankizumab (150 mg, at weeks 0, 4, and 16)	407	0	100	0
Reich et al., 2017 (58)	reSURFACE 2/ NCT01729754	Tildrakizumab (100 mg, at weeks 0, 4, and 16) Tildrakizumab (200 mg, at weeks 0, 4, and 16)	307 314	0 1	156	0

**Table 4:** Cases of tuberculosis, hepatitis, and herpes zoster reported in the OLE studies.

Source	Trial Name/ClinicalTrials.gov identifier	Treatment (dose)	Duration of Open-label Extension Period	Case of Tuberculosis	Case of Hepatitis	Case of Herpes zoster
Lebwohl et al., 2015 (17)	AMAGINE-2/NCT01708603	Brodalumab (140mg or 210 mg, Q2W)	52 weeks	NA	1	1
Lebwohl et al., 2019 (62)	NCT01101100	Brodalumab (210 mg, Q2W)	6 years	1	1	NA
van der Heijde et al., 2018 (64)	SPIRIT-P1/NCT01695239	Ixekizumab (160 mg at week 0, then 80 mg Q2W) Ixekizumab (160 mg at week 0, then 80 mg Q4W)	52 weeks	0 0	0 1	1 0
Chandran et al., 2020 (65)	SPIRIT-P1/NCT01695239	Ixekizumab (160 mg at week 0, then 80 mg Q2W) Ixekizumab (160 mg at week 0, then 80 mg Q4W)	3 years	1 0	NA NA	1 0
Genovese et al., 2018 (66)	SPIRIT-P2/NCT02349295	Ixekizumab (160 mg at week 0, then 80 mg Q2W) Ixekizumab (160 mg at week 0, then 80 mg Q4W)	52 weeks	0 1	0 0	2 0
Gordon et al., 2014 (67)	NCT01107457	Ixekizumab (6 doses at 0, 2, 4, 8, 12, and 16 weeks, then 120 mg Q4W)	52 weeks	0	NA	NA
Blauvelt et al., 2017 (69)	CLEAR/ NCT02074982	Secukinumab (300 mg, QW for 4 weeks, then Q4W)	52 weeks	0	NA	NA
McInnes et al., 2017 (70)	FUTURE2/NCT01752634	Secukinumab (75, 150 or 300 mg, QW for 4 weeks, then Q4W)	104 weeks	0	NA	NA
van der Heijde et al., 2020 (71)	FUTURE5/NCT02404350	Secukinumab (150 mg, QW for 4 weeks, then Q4W) Secukinumab (300 mg, QW for 4 weeks, then Q4W)	52 weeks	0 0	NA NA	NA NA
Bissonnette et al., 2018 (73)	SCULPTURE/NCT01640951	Secukinumab (300mg, QW for 4 weeks, then Q4W, fixed interval)	1 year 2 years 3 years 4 years 5 years	0 0 0 0 0	0 0 0 0 0	0 0 0 0 0
Coates et al., 2022 (75)	BE ACTIVE/NCT03347110	Bimekizumab (160 mg Q4W); Bimekizumab (320 mg Q4W)	152 weeks	0	NA	0
Strober et al., 2023 (76)	BE RADIANT/ NCT03536884	Bimekizumab (320 mg Q4W)	1 year 2 years	0 0	NA NA	NA NA
McInnes et al., 2021 (79)	DISCOVER-2/ NCT03158285	Guselkumab (100 mg, Q4W) Guselkumab (100 mg, at weeks 0 and 4, then Q8W)	52 weeks	0 0	1 0	0 0
McInnes et al., 2022 (80)	DISCOVER-2/ NCT03158285	Guselkumab (100 mg, Q4W) Guselkumab (100 mg, at weeks 0 and 4, then Q8W)	112 weeks	0 0	1 0	0 1
Gooderham et al., 2022 (81)	UltIMMa-1/NCT02684370; UltIMMa-2/NCT02684357	Risankizumab (150 mg, at weeks 0 and 4, then Q12W)	52 weeks	0	NA	NA
Gooderham et al., 2022 (81)	LIMMitless/NCT03047395	Risankizumab (150 mg, at weeks 0 and 4, then Q12W)	172 weeks	0	NA	NA
Papp et al., 2021 (82)	LIMMitless/NCT03047395	Risankizumab (150 mg, at weeks 0 and 4, then Q12W)	208 weeks	0	NA	NA
Reich et al., 2020 (83)	reSURFACE 1/ NCT01722331; reSURFACE 2/ NCT01729754	Tildrakizumab (100 mg, at weeks 0 and 4, then Q12W) Tildrakizumab (200 mg, at weeks 0 and 4, then Q12W)	148 weeks	0 0	NA NA	0 1
Thaci et al., 2021 (84)	reSURFACE 1/ NCT01722331; reSURFACE 2/ NCT01729754	Tildrakizumab (100 mg, at weeks 0 and 4, then Q12W) Tildrakizumab (200 mg, at weeks 0 and 4, then Q12W)	256 weeks	0 0	NA NA	0 1

### 3.2 Short-term risk with IL-17 inhibitors

#### 3.2.1 Serious infection

**Figure 2:**
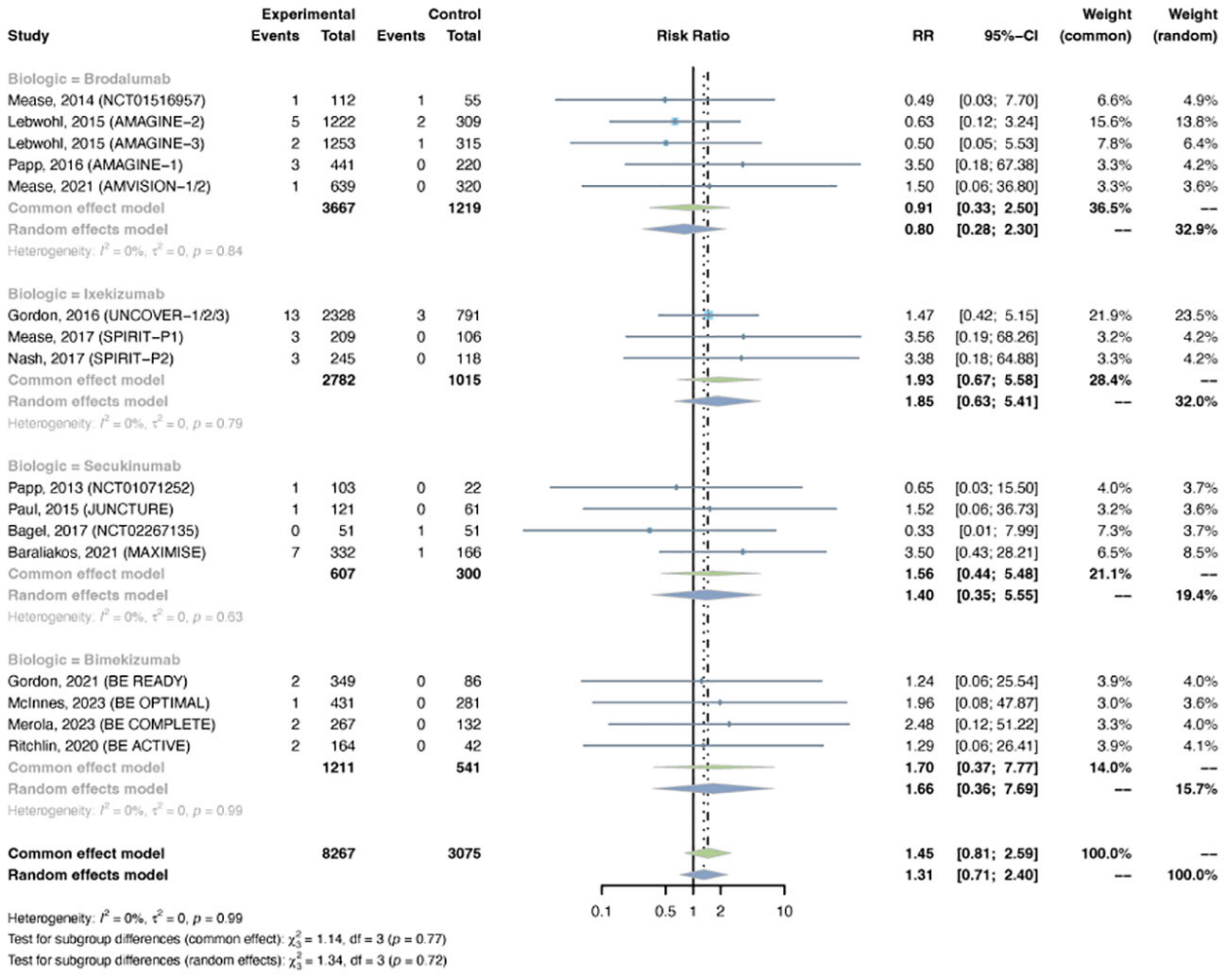
Pooled risk ratio (RR) of serious infection with the treatment of IL-17 inhibitors vs placebo.

Serious infections were reported in 16 trials, including 8,267 patients in the treatment group and 3,075 patients in the placebo group. As there was no evidence of statistical heterogeneity between trials (I^2^ = 0.0%, P-value = 0.94), a common effects model was fit. The overall RR of serious infection was not increased with IL-17 inhibitors (RR = 1.45, 95% CI: 0.81-2.59) (**Figure 2**). The RR was not increased with brodalumab (RR = 0.91, 95% CI: 0.33-2.50), ixekizumab (RR = 1.93, 95% CI: 0.67-5.58), secukinumab (RR = 1.56, 95% CI: 0.44-5.48), or bimekizumab (RR = 1.70, 95% CI: 0.37-7.77). No subgroup differences were found among the four IL-17 antagonists (P-value = 0.77). There was no increase in the RR of severe infection in patients with PsO (RR = 1.08, 95% CI: 0.52-2.26) or PsA (RR = 2.20, 95% CI: 0.84-5.76) (**Figure S2**). No subgroup differences were found among the two indications (P-value = 0.25).

#### 3.2.2 Overall infection

**Figure 3:**
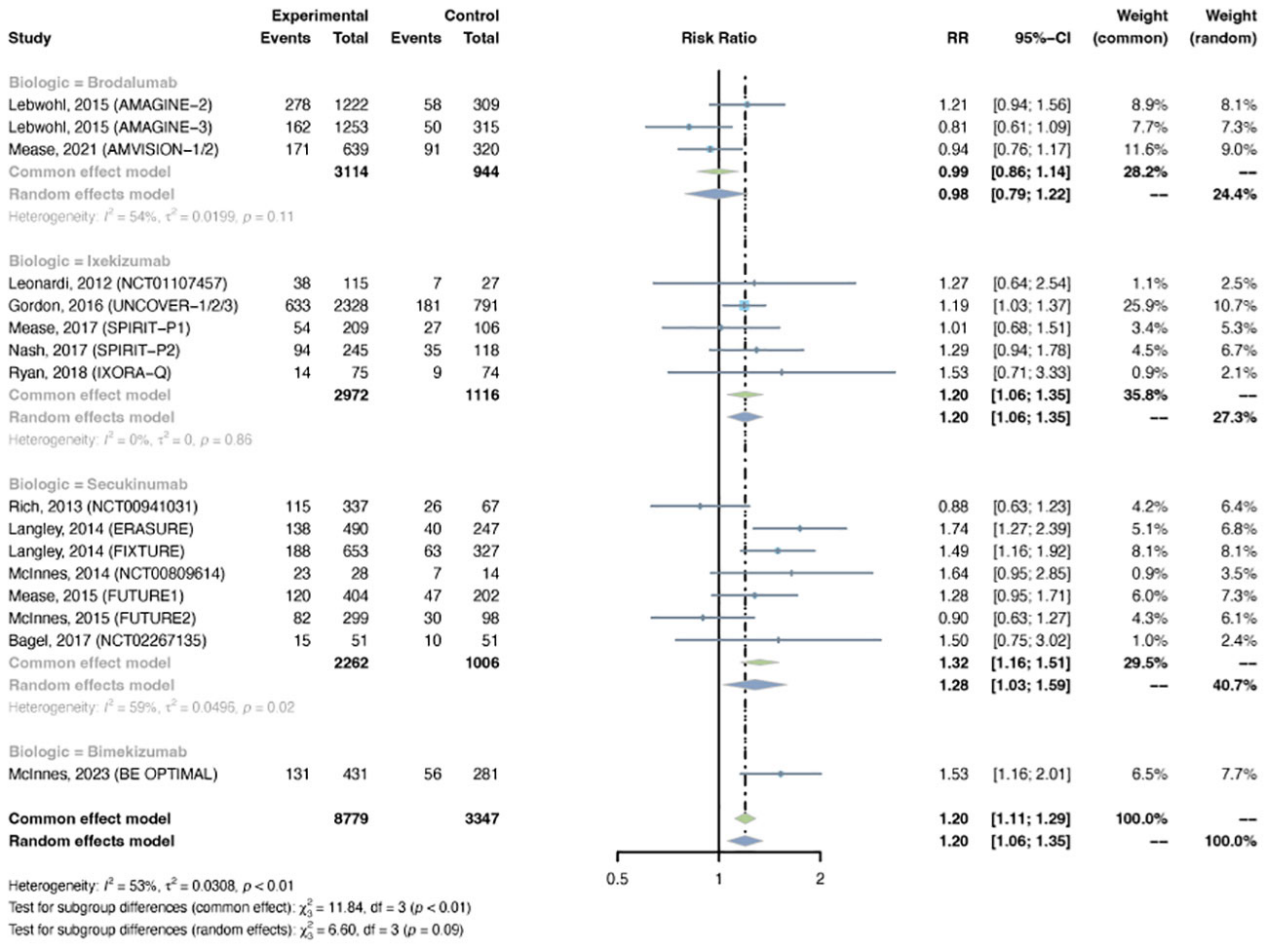
Pooled risk ratio (RR) of overall infection with the treatment of IL-17 inhibitors vs placebo.

Sixteen trials were selected, with 8,779 patients in the treatment arm and 3,347 patients in the placebo arm. There was major between-trial heterogeneity (I^2^ = 53%, P-value < 0.01), so a random effects model was used. Overall, the pooled RR of overall infection in patients with psoriasis receiving IL-17 inhibitors was 1.20 (95% CI: 1.06-1.35) (**Figure 3**). The RR was increased with ixekizumab (RR = 1.20, 95% CI: 1.06-1.35), secukinumab (RR = 1.28, 95% CI: 1.03-1.33), and bimekizumab (RR = 1.53, 95% CI: 1.16-2.01), whereas it was not increased with brodalumab (RR = 0.98, 95% CI: 0.79-1.22). The pooled RRs were 1.22 (95% CI: 1.01-1.46) in PsO, and 1.17 (95% CI: 0.99-1.39) in PsA (**Figure S3**).

#### 3.2.3 Malignancy

**Figure 4:**
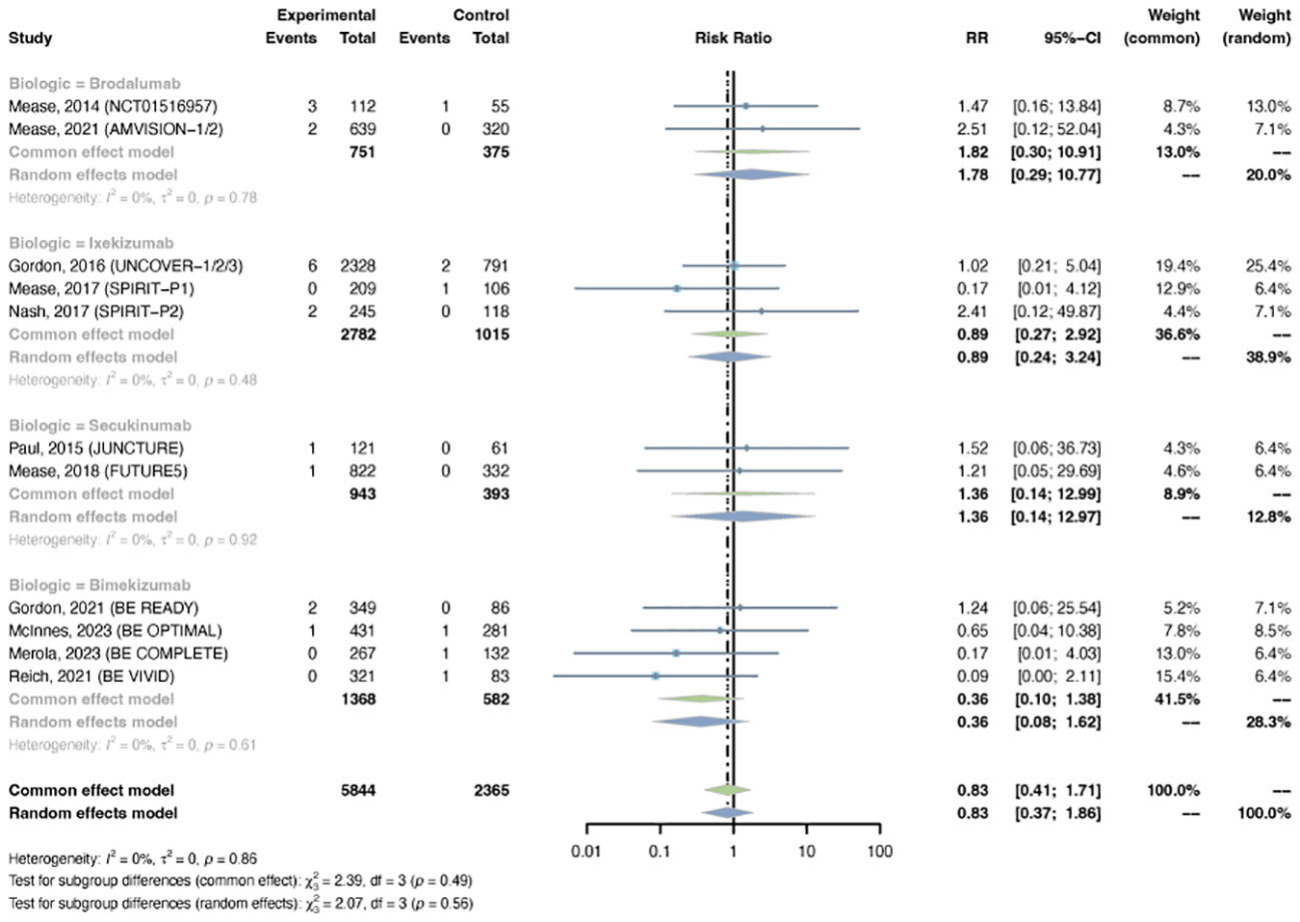
Pooled risk ratio (RR) of malignancy with the treatment of IL-17 inhibitors vs placebo.

The outcomes of malignancy were reported in 11 RCTs, including 5,844 patients in the treatment group and 2,365 patients in the placebo group. A common-effects model was used based on the low heterogeneity between trials (I^2^= 0%, P-value = 0.86). The overall RR of malignancy was not increased in psoriasis patients using IL-17 inhibitors (RR = 0.83, 95% CI: 0.41-1.71) (**Figure 4**). The RR was not increased with brodalumab (RR = 1.82, 95% CI: 0.30-10.91), ixekizumab (RR = 0.89, 95% CI: 0.27-2.92), secukinumab (RR = 1.36, 95% CI: 0.14-12.99), or bimekizumab (RR = 0.36, 95% CI: 0.10-1.38). No subgroup differences were found among the different anti-IL-17 agents (P-value = 0.49). The pooled RR of malignancy was not increased in both PsO (RR = 0.77, 95% CI: 0.26-2.28) and PsA (RR = 0.88, 95% CI: 0.34-2.32) (**Figure S4**). No subgroup differences were found among the two conditions (P-value = 0.85).

#### 3.2.4 Nasopharyngitis

**Figure 5:**
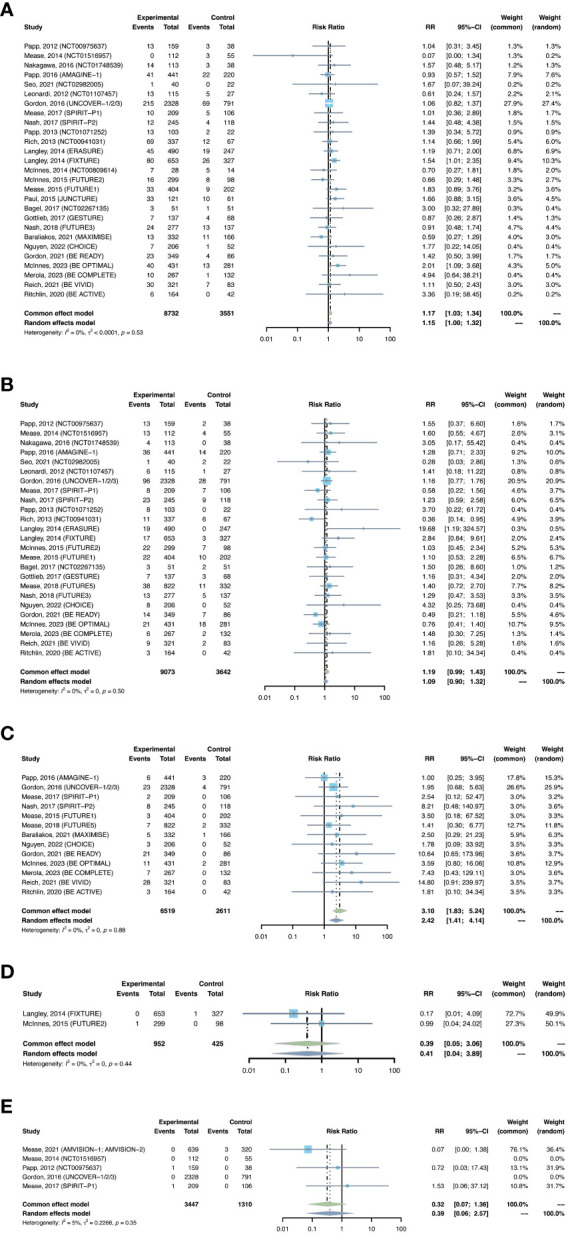
Pooled risk ratio (RR) of **(A)** nasopharyngitis; **(B)** upper respiratory tract infection; **(C)** *Candida* infection; **(D)** hepatitis; **(E)** herpes zoster with the treatment of IL-17 inhibitors vs placebo.

The outcomes of nasopharyngitis were reported in 27 trials, including 8,732 patients in the treatment group and 3,551 patients in the placebo group. The overall pooled RR of nasopharyngitis in patients receiving IL-17 inhibitors was 1.17 (95% CI: 1.03-1.34) (**Figure 5A**). Subgroup analyses by biologic showed increased RRs of nasopharyngitis with secukinumab (RR = 1.21, 95% CI: 1.00-1.48) and bimekizumab (RR = 1.76, 95% CI: 1.16-2.67), instead of brodalumab and ixekizumab (**Figure S5A**). The RR of nasopharyngitis was increased in PsO, but not in PsA (**Figure S5B**).

#### 3.2.5 Upper respiratory tract infection

The outcomes of upper respiratory tract infection were reported in 25 trials, including 9,073 patients in the treatment group and 3,642 patients in the placebo group. The overall RR of upper respiratory tract infection was found to be 1.19 (95% CI: 0.99-1.43) (**Figure 5B**). The risk of upper respiratory tract infection was increased with secukinumab (RR = 1.43, 95% CI: 1.05-1.96), but not with the other three drugs (**Figure S6A**). The RR of upper respiratory tract infection was not increased in either PsO or PsA (**Figure S6B**).

#### 3.2.6 *Candida* infection

The outcomes of *Candida* infection were reported in 13 trials, including 6,519 patients in the treatment group and 2,611 patients in the placebo group. The overall RR of *Candida* infection was 3.10 (95% CI: 1.83-5.24) (**Figure 5C**). The risk of *Candida* infection was increased with ixekizumab (RR = 2.59, 95% CI: 1.02-6.53), and was significantly increased with bimakizumab (RR = 6.47, 95% CI: 2.29-18.33) (**Figure S7A**). The RR of *Candida* infection was increased in both PsA and in PsO (**Figure S7B**).

#### 3.2.7 Tuberculosis

There were 12 studies reporting the outcomes of tuberculosis, including 3,829 patients in the treatment group and 1,587 patients in the placebo group. In all the included RCTs, there was no case of latent or active tuberculosis reported in both treatment and placebo groups (**Table 3**). Therefore, quantitative meta-analysis was not performed. Nevertheless, we could obviously observe a quite low short-term risk of tuberculosis with IL-17 inhibitors in population without history or evidence of tuberculosis.

#### 3.2.8 Hepatitis

There were 2 studies reporting the outcomes of hepatitis with IL-17 inhibitors, including 952 patients in the treatment group and 425 patients in the placebo group. There were only one case of hepatitis C reported in the treatment groups (30), and one case of hepatitis B reported in the placebo groups (28) (**Table 3**). Generally, the incidence of hepatitis was very low in the induction periods. Meta-analysis also did not reveal an increased risk (RR = 0.39, 95% CI: 0.05-3.06) (**Figure 5D**). Subgroup analysis was not performed due to limited data.

#### 3.2.9 Herpes zoster

There were 2 studies reporting the outcomes of herpes zoster, including 3,447 patients in the treatment group and 1,310 patients in the placebo group. The overall RR of herpes zoster was 0.32 (95% CI: 0.07-1.36) (**Figure 5E**). As shown in **Table 3**, the overall incidence of herpes zoster was low. Two cases were reported in the treatment group, and three cases were reported in the placebo group. Subgroup analysis was not performed due to limited data.

### 3.3 Long-term incidence rates with IL-17 inhibitors

#### 3.3.1 Serious infection

**Figure 6:**
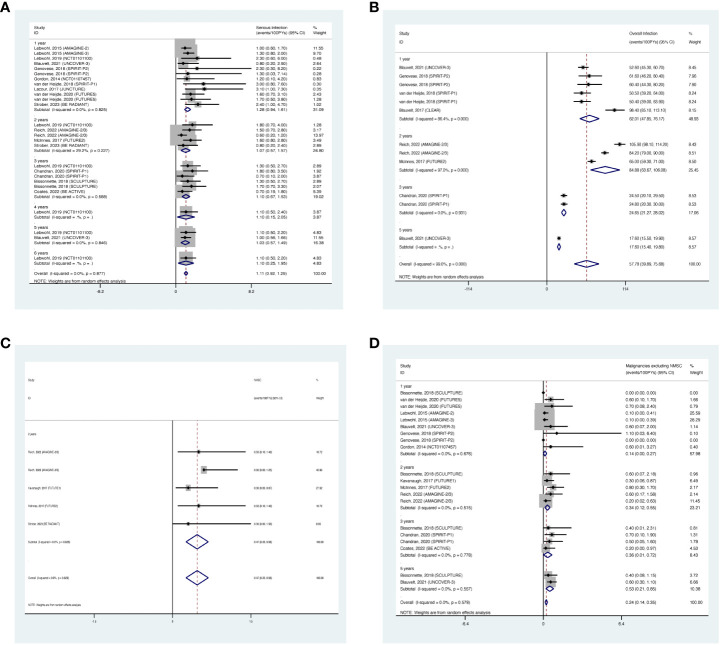
Pooled exposure-adjusted incidence rates (EAIRs) of **(A)** serious infection; **(B)** overall infection; **(C)** nonmelanoma skin cancer (NMSC); and **(D)** malignancies excluding NMSC in psoriasis patients receiving long-term anti-IL-17 treatment.

The overall long-term EAIR of serious infection was 1.11/100 PYs (95% CI: 0.92-1.29) in patients with psoriatic disease using IL-17 inhibitors (**Figure 6A**). The EAIR of serious infection was 1.28/100 PYs for 1 year, 1.07/100 PYs for 2 years, 1.10/100 PYs for 3 years, 1.10/100 PYs for 4 years, 1.03/100 PYs for 5 years, and 1.10/100 PYs for 6 years. No evidence of an increased incidence rate of serious infection was found with a longer duration of anti-IL-17 treatment.

#### 3.3.2 Overall infection

The overall long-term EAIR of overall infection was 57.78/100 PYs (95% CI: 39.89-75.68) (**Figure 6B**). The EAIR of overall infection was 62.01/100PYs for 1 year, 84.88/100 PYs for 2 years, 24.65/100 PYs for 3 years, and 17.60/100 PYs for 5 years, respectively. There was no evidence showing an increase in the incidence rate of overall infection with the prolonged duration of anti-IL-17 treatment.

#### 3.3.3 NMSC

The overall long-term EAIR of NMSC was 0.47/100PYs (95% CI: 0.26-0.68) in psoriasis patients receiving IL-17 inhibitors (**Figure 6C**). The data were limited to 2-year outcomes.

#### 3.3.4 Malignancies excluding NMSC

The overall long-term EAIR of malignancies excluding NMSC was 0.24/100 PYs (95% CI: 0.14-0.35) in psoriasis patients receiving IL-17 inhibitors (**Figure 6D**). The EAIR of malignancies excluding NMSC was 0.14/100PYs for 1 year, 0.34/100PYs for 2 years, 0.36/100PYs for 3 years, and 0.53/100PYs for 5 years. No evidence of significantly increased incidence rate of malignancies excluding NMSC was found with longer duration.

#### 3.3.5 Nasopharyngitis

**Figure 7:**
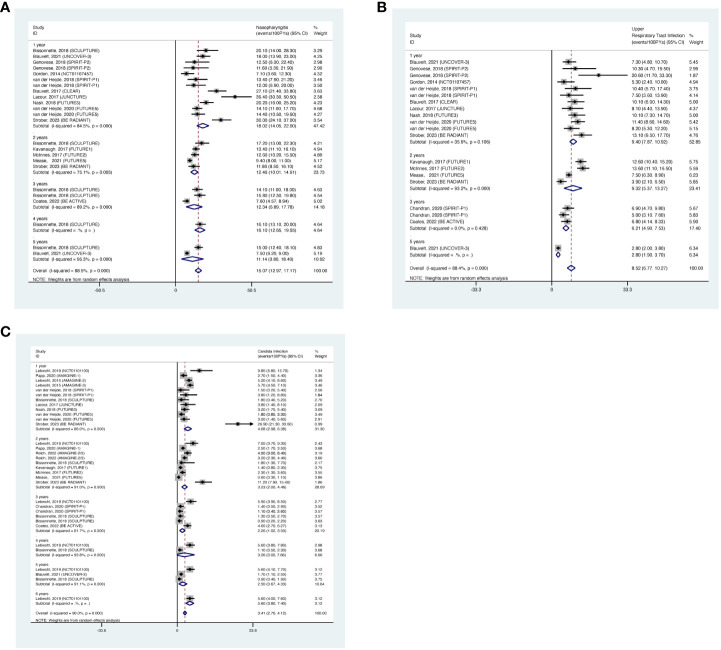
Pooled exposure-adjusted incidence rates (EAIRs) of **(A)** nasopharyngitis; **(B)** upper respiratory tract infection; **(C)** *Candida* infection in psoriasis patients receiving long-term anti-IL-17 treatment.

The overall long-term EAIR of nasopharyngitis was 15.07/100PYs (95% CI: 12.97-17.17) in psoriasis patients receiving IL-17 inhibitors (**Figure 7A**). The EAIR of nasopharyngitis was 18.02/100PYs for 1 year, 12.46/100PYs for 2 years, 12.34/100PYs for 3 years, 16.10/100PYs for 4 years, and 11.14/100PYs for 5 years. No evidence of an increased incidence rate of nasopharyngitis was found with longer duration.

#### 3.3.6 Upper respiratory tract infection

The overall long-term EAIR of upper respiratory tract infection was 8.52/100PYs (95% CI: 6.77-10.27) (**Figure 7B**). The EAIR of upper respiratory tract infection was 9.40/100PYs for 1 year, 9.32/100PYs (95% CI: 6.90-15.33) for 2 years, 6.21/100PYs for 3 years, and 2.80/100PYs for 5 years. No evidence of an increased incidence rate of upper respiratory tract infection was found with the prolonged duration.

#### 3.3.7 *Candida* infection

The overall long-term EAIR of *Candida* infection was 3.41/100PYs (95% CI: 2.70-4.12) in psoriasis patients receiving IL-17 inhibitors (**Figure 7C**). The EAIR of *Candida* infection was 4.68/100PYs for 1 year, 3.23/100PYs for 2 years, 2.26/100PYs for 3 years, 3.26/100PYs for 4 years, 2.50/100PYs for 5 years, and 5.60/100PYs for 6 years. No evidence of an increased incidence rate of *Candida* infection was found with the prolonged duration.

#### 3.3.8 Tuberculosis

There were 12 studies reporting the long-term incidence of tuberculosis. One case of latent tuberculosis was reported in the six-year OLE period of brodalumab (210 mg, every 2 weeks) (62); one case was reported in the three-year OLE period of Ixekizumab (160 mg at week 0, then 80mg every 2 weeks) (65); and one case was reported in the one-year OLE period of Ixekizumab (160 mg at week 0, then 80mg every 4 weeks) (66). Other included studies all reported 0/100PYs of tuberculosis. No active or reactivated tuberculosis cases were ever reported in all the included studies. No quantitative meta-analysis was performed due to the limited data. In general, the long-term EAIRs were quite low.

#### 3.3.9 Hepatitis

There were 5 studies reporting the long-term incidence of hepatitis. One case of hepatitis was reported in the one-year and six-year OLE periods of brodalumab (210 mg, every 2 weeks) (17); and one case of hepatitis B was reported in the one-year OLE period of ixekizumab (160 mg at week 0, then 80mg every 4 weeks) (64). No cases of hepatitis were reported by other included studies. No quantitative meta-analysis was performed due to the limited data.

#### 3.3.10 Herpes zoster

There were 6 studies reporting the long-term incidence of herpes zoster. One case of herpes zoster was reported in the one-year OLE period of brodalumab (210 mg, every 2 weeks) (17); one case in the one-year and three-year OLE periods of ixekizumab (160 mg at week 0, then 80mg every 2 weeks) (64, 65); 2 in the one-year period of ixekizumab (160 mg at week 0, then 80mg every 2 weeks) (66). Other included studies reported 0/100PYs of EAIRs though the whole trials. No quantitative meta-analysis was performed due to the limited data. In general, the EAIRs were very low.

### 3.4 Short-term risk with IL-23 inhibitors

#### 3.4.1 Serious infection

**Figure 8:**
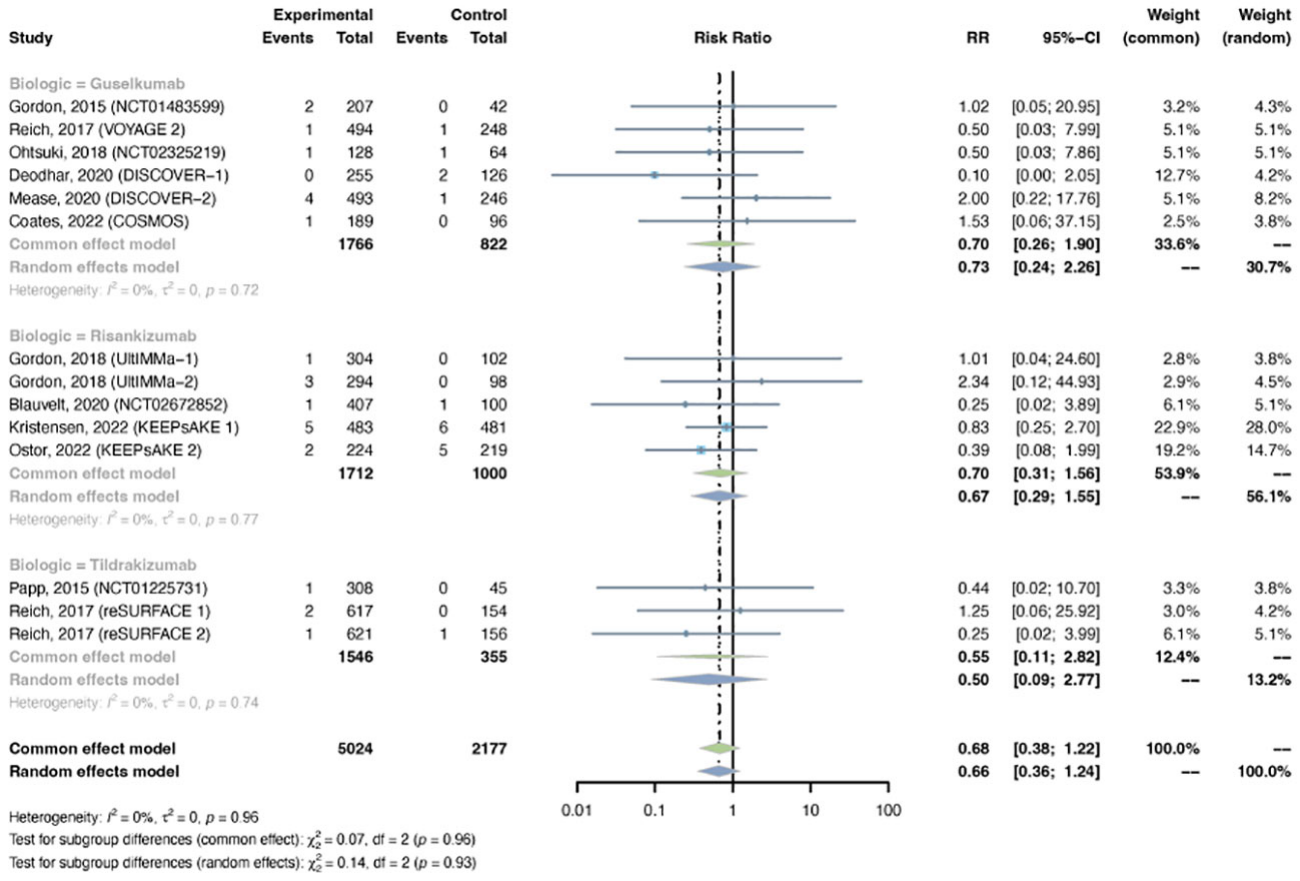
Pooled risk ratio (RR) of serious infection with the treatment of IL-23 inhibitors vs placebo.

Serious infections were reported in 14 trials, including 5,024 patients in the treatment group and 2,177 patients in the placebo group. A common-effects model was adapted based on low heterogeneity (I^2^ = 0%, p = 0.96). The overall RR of serious infection was not increased with IL-23 inhibitors (RR = 0.68, 95% CI: 0.38-1.22) (**Figure 8**). The pooled RR was 0.70 (95% CI: 0.26-1.90) with guselkumab, 0.70 (95% CI: 0.31-1.56) with risankizumab, and 0.55 (95% CI: 0.11-2.82) with tidrakizumab. No subgroup differences were found among the three anti-IL-23 agents (P-value = 0.96). The pooled RR was not increased in either PsO (RR = 0.70, 95% CI: 0.28-1.76) or PsA (RR = 0.67, 95% CI: 0.31-1.42) (**Figure S8**). No subgroup differences were found among the two conditions (P-value = 0.94).

#### 3.4.2 Overall infection

**Figure 9:**
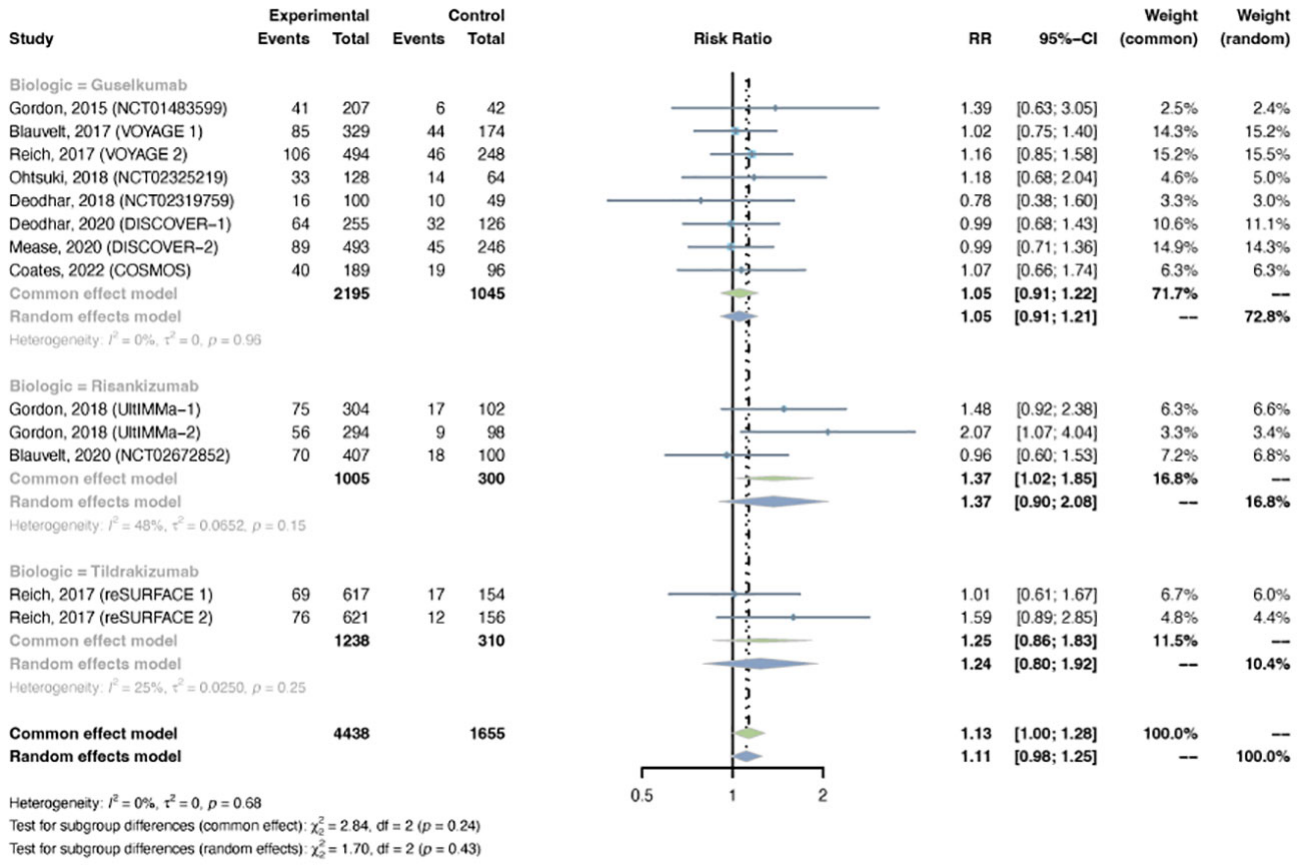
Pooled risk ratio (RR) of overall infection with the treatment of IL-23 inhibitors vs placebo.

The outcomes of overall infection were reported in 13 trials, including 4,438 patients in the treatment group and 1,655 patients in the placebo group. The overall RR of overall infection in psoriasis patients using IL-23 inhibitors was 1.13 (95% CI: 1.00-1.28) (**Figure 9**). Statistical heterogeneity was low between trials (I^2^ = 0%, P-value = 0.68). The pooled RR of overall infection was moderately increased with risankizumab (RR = 1.37, 95% CI: 1.02-1.85), instead of guselkumab (RR = 1.05, 95% CI: 0.91-1.22) or tidrakizumab (RR = 1.25, 95% CI: 0.86-1.83). The pooled RR was increased in PsO (RR = 1.21, 95% CI: 1.04-1.41), but not in PsA (RR = 0.98, 95% CI: 0.80-1.21) (**Figure S9**).

#### 3.4.3 Malignancy

**Figure 10:**
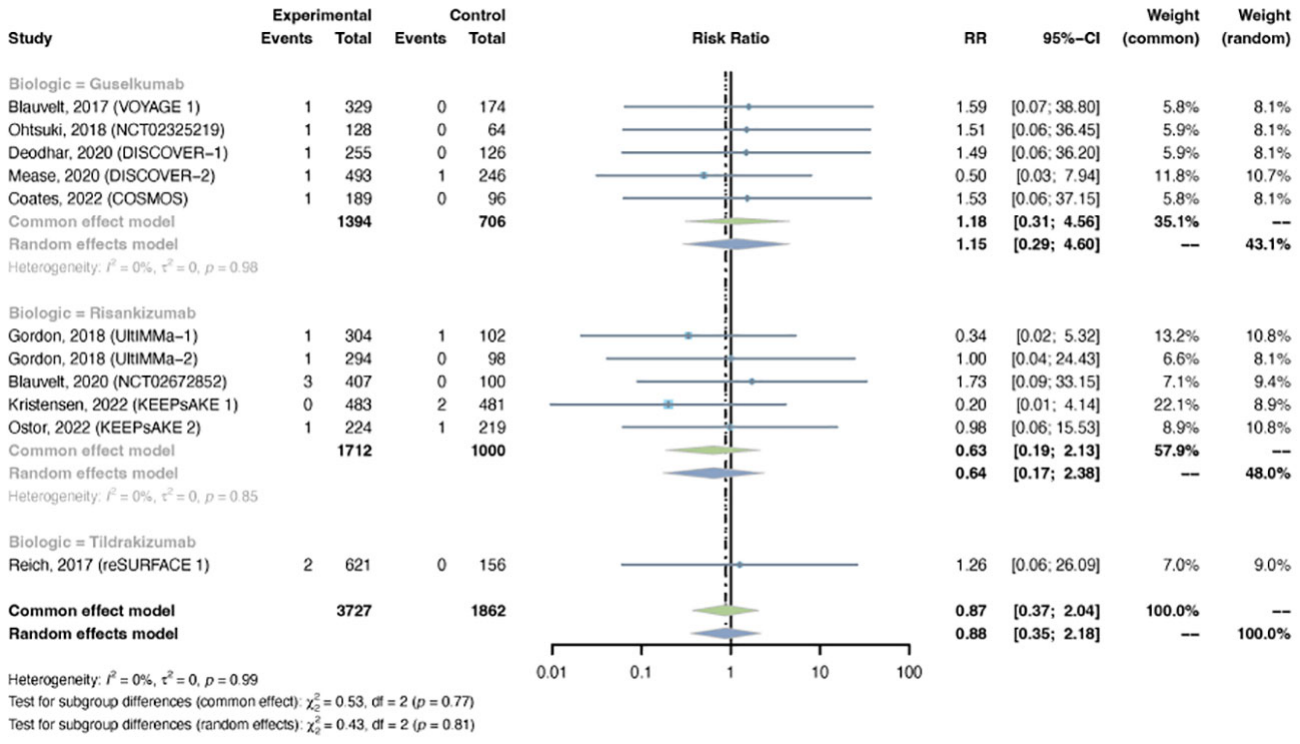
Pooled risk ratio (RR) of malignancy with the treatment of IL-23 inhibitors vs placebo.

The outcomes of malignancy were reported in 11 trials, including 3,727 patients in the treatment group and 1,862 patients in the placebo group. No evidence of statistical heterogeneity between trials was found (I^2^ = 0.0%, P-value = 0.99), so a common-effects model was adapted. The pooled RR of malignancy in psoriasis patients with IL-23 inhibitors was not increased (RR = 0.87, 95% CI: 0.37-2.04) (**Figure 10**). The RR of malignancy was not increased with either guselkumab (R = 1.18, 95% CI: 0.31-4.56), risankizumab (RR = 0.63, 95% CI: 0.19-2.13), or tidrakizumab (RR = 1.26, 95% CI: 0.06-26.09). No subgroup differences were found among the three anti-IL-23 agents (P-value = 0.77). The pooled RR was not increased in PsO (RR = 1.10, 95% CI: 0.33-3.66) or PsA (RR = 0.67, 95% CI: 0.19-2.33) (**Figure S10**). No subgroup differences were found among the two conditions (P-value = 0.57).

#### 3.4.4 Nasopharyngitis

**Figure 11:**
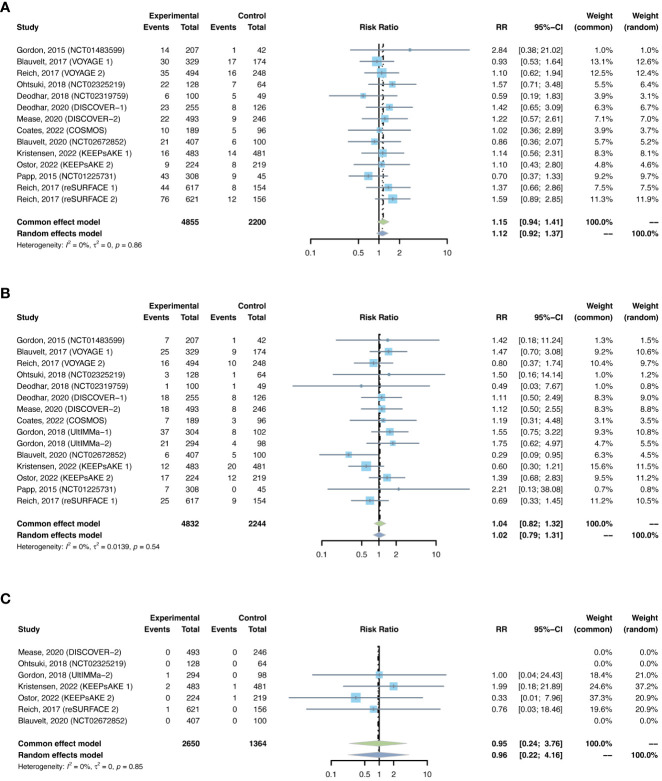
Pooled risk ratio (RR) of **(A)** nasopharyngitis; **(B)** upper respiratory tract infection; **(C)** herpes zoster with the treatment of IL-23 inhibitors vs placebo.

The outcomes of nasopharyngitis were reported in 14 studies, including 4,855 patients in the treatment group and 2,200 in the placebo group. The overall RR of nasopharyngitis was not increased with IL-23 inhibitors (RR = 1.15, 95% CI: 0.94-1.41) (**Figure 11A**). Subgroup analysis by biologic showed that none of the anti-IL-23 agents could improve the risk of nasopharyngitis (**Figure S11A**). Subgroup analysis by indications suggested that the risk of nasopharyngitis was not increased in either PsO or PsA (**Figure S11B**).

#### 3.4.5 Upper respiratory tract infection

The outcomes of upper respiratory tract infection were reported in 15 studies, including 4,832 patients in the treatment group and 2,244 patients in the placebo group. The overall RR of upper respiratory tract infection was not increased with IL-23 inhibitors (RR = 1.04, 95% CI: 0.82-1.32) (**Figure 11B**). Subgroup analysis by biologic showed that none of the anti-IL-23 agents could improve the risk of upper respiratory tract infection (**Figure S12A**). Subgroup analysis by indications suggested that the risks of upper respiratory tract infection were not increased in either PsO or PsA (**Figure S12B**).

#### 3.4.6 Tuberculosis

There were 14 studies reporting the outcomes of tuberculosis, including 4,935 patients in the treatment group and 2,130 patients in the placebo group. Similar to IL-17 inhibitors, there was zero case of latent or active tuberculosis reported in both treatment and placebo groups with IL-23 inhibitors (**Table 3**). As a result, quantitative meta-analysis was not performed. A very low short-term risk of tuberculosis with IL-23 inhibitors was reported in population without history or evidence of tuberculosis.

#### 3.4.7 Hepatitis

There were 2 studies reporting the outcomes of hepatitis with IL-23 inhibitors, including 621 patients in the treatment group and 310 patients in the placebo group. There were only one case of hepatitis B reported in the treatment groups (50) (**Table 3**). Meta-analysis was not performed due to the limited data. Generally, the incidence of hepatitis was found very low in the induction periods.

#### 3.4.8 Herpes zoster

There were 7 studies reporting the outcomes of herpes zoster, including 2,650 patients in the treatment group and 1,364 patients in the placebo group. Quantitative meta-analysis suggested that the overall RR of herpes zoster was not increased with IL-23 inhibitors (RR = 0.95, 95% CI: 0.24-3.76) (**Figure 11C**). Subgroup analysis was not performed due to limited data.

### 3.5 Long-term incidence rates with IL-23 inhibitors

#### 3.5.1 Serious infection

**Figure 12:**
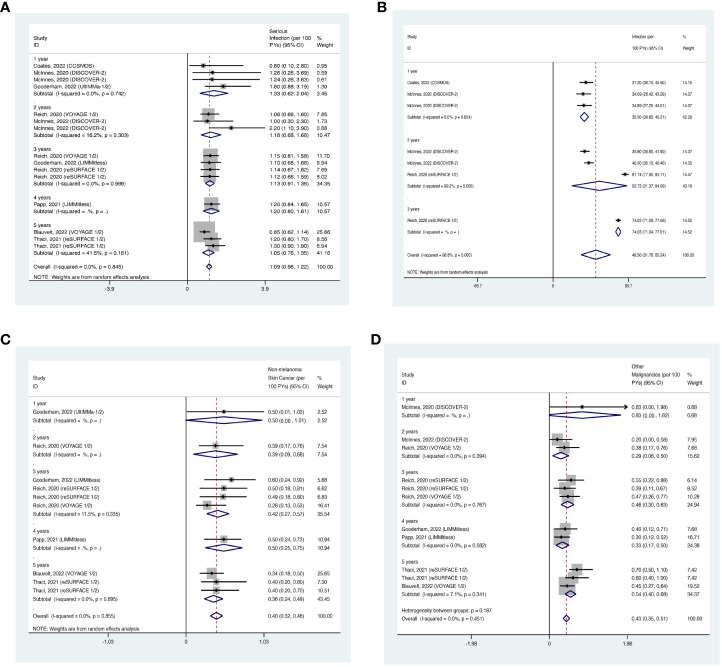
Pooled exposure-adjusted incidence rates (EAIRs) of **(A)** serious infection; **(B)** overall infection; **(C)** nonmelanoma skin cancer (NMSC); and **(D)** malignancies excluding NMSC in psoriasis patients receiving long-term anti-IL-23 treatment.

The overall long-term EAIR of serious infection was 1.09/100 PYs (95% CI: 0.96-1.22) in patients with psoriatic disease using IL-23 inhibitors (**Figure 12A**). The EAIR of serious infection was 1.33/100 PYs for 1 year, 1.18/100 PYs for 2 years, 1.13/100 PYs for 3 years, 1.20/100 PYs for 4 years, and 1.05/100 PYs for 5 years. No evidence of an increased incidence rate of serious infection was found with the prolonged duration of anti-IL-23 treatment.

#### 3.5.2 Overall infection

The overall long-term EAIR of overall infection was 48.50/100 PYs (95% CI: 31.76-65.24) (**Figure 12B**). The EAIR of overall infection was 35.50/100PYs in psoriasis patients receiving IL-17 inhibitors for 1 year, 52.73/100 PYs for 2 years, 74.03/100 PYs for 3 years. There was also no evidence showing an increased incidence rate of overall infection with the prolonged duration of anti-IL-23 treatment.

#### 3.5.3 NMSC

The overall long-term EAIR of NMSC was 0.40/100 PYs (95% CI: 0.32-0.48) in psoriasis patients receiving IL-23 inhibitors (**Figure 12C**). The EAIR of NMSC was 0.50/100PYs in psoriasis patients receiving IL-23 inhibitors for 1 year, 0.39/100PYs for 2 years, 0.42/100PYs for 3 years, 0.50/100PYs for 4 years, and 0.36/100PYs for 5 years. No evidence of an increased incidence rate of NMSC was found with the prolonged treatment duration.

#### 3.5.4 Malignancies excluding NMSC

The overall long-term EAIR of malignancies excluding NMSC was 0.43/100 PYs (95% CI: 0.35-0.51) in psoriasis patients receiving IL-23 inhibitors (**Figure 12D**). The EAIR of malignancies excluding NMSC was 0.83/100PYs for 1 year, 0.29/100PYs for 2 years, 0.46/100PYs for 3 years, 0.33/100PYs for 4 years, and 0.54/100PYs for 5 years. No evidence of an increased incidence rate of malignancies excluding NMSC was found with the prolonged duration.

#### 3.5.5 Nasopharyngitis

**Figure 13:**
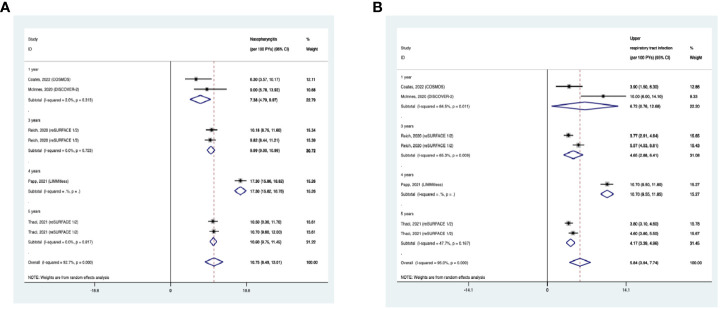
Pooled exposure-adjusted incidence rates (EAIRs) of **(A)** nasopharyngitis; **(B)** upper respiratory tract infection in psoriasis patients receiving long-term anti-IL-23 treatment.

The overall long-term EAIR of nasopharyngitis was 10.75/100PYs (95% CI: 8.49-13.01) in psoriasis patients receiving IL-23 inhibitors (**Figure 13A**). The EAIR of nasopharyngitis was 7.38/100PYs for 1 year, 9.99/100PYs for 3 years, 17.30/100PYs for 4 years, and 10.60/100PYs for 5 years. No evidence of an increased incidence rate of nasopharyngitis was found with the prolonged treatment duration.

#### 3.5.6 Upper respiratory tract infection

The overall long-term EAIR of upper respiratory tract infection was 5.84/100PYs (95% CI: 3.94-7.74) (**Figure 13B**). The EAIR of upper respiratory tract infection was 6.72/100PYs for 1 year, 4.65/100PYs for 3 years, 10.70/100PYs for 5 years, and 4.17/100PYs. No evidence of an increased incidence rate of upper respiratory tract infection was found with the longer duration.

#### 3.5.7 Tuberculosis

There were 6 studies reporting the long-term incidence of tuberculosis. Of all the included studies, the long-term EAIRs of tuberculosis were 0/100PYs, with no case of active, reactivated or latent tuberculosis reported throughout the whole trials. Therefore, the overall EAIR of tuberculosis with IL-23 inhibitors was estimated to be 0/100PYs. No quantitative meta-analysis was performed due to the limited data.

#### 3.5.8 Hepatitis

There was 1 study reporting the long-term incidence of hepatitis. Only one case of hepatitis B was reported in the two-year OLE period of guselkumab (100 mg, every 4 weeks) (80). No case of hepatitis with other types of IL-23 inhibitors was reported. No quantitative meta-analysis was performed due to the limited data.

#### 3.5.9 Herpes zoster

There were 4 studies reporting the long-term incidence of herpes zoster. One case in the two-year OLE period of guselkumab (100 mg, every 4 weeks) (80), one in the three-year and five-year OLE periods of Tildrakizumab (200 mg, at week 4 and every 12 weeks thereafter) (83, 84). Other included studies reported 0/100PYs of EAIRs though the whole trials. No quantitative meta-analysis was performed due to the limited data. In general, the EAIRs were very low.

### 3.6 Publication bias and sensitivity analyses

No evidence of publication bias was found with “peters” tests for serious infection (P-value = 0.66), overall infection (P-value = 0.62), malignancy (P-value = 0.82), nasopharyngitis (P-value = 0.73), upper respiratory tract infection (P-value = 0.98), or *Candida* infection (P-value = 0.06) with IL-17 inhibitors; serious infection (P-value = 0.63), overall infection (P-value = 0.89), malignancy (P-value = 0.22), nasopharyngitis (P-value = 0.65), or upper respiratory tract infection (P-value = 0.08) with IL-23 inhibitors. Funnel plots created for all of the above outcomes were found to be symmetrical (**Figures S13**, **S14**). Sensitivity analyses suggested that omitting any one of the studies had little effect on the final results in general (**Figures S15**–**S18**).

## 4 Discussion

Therapeutic agents that specifically target the IL-17 family and IL-23 have demonstrated excellent efficacy in PsO and PsA (5, 6). However, the potential for infection and malignancy has been a concern with respect to the therapies which suppress immune pathways (9, 85–87). Individual studies are not large enough in sample sizes or of long enough duration to ascertain uncommon adverse events. Hence, we performed a meta-analysis of RCTs and OLE studies to estimate the risk and incidence of infections and malignancies with treatments of IL-17 or IL-23 inhibitors. The results suggested that anti-IL-17 or anti-IL-23 treatments did not increase the short-term risk of serious infection or malignancy in adult patients with psoriatic disease. Correspondingly, the long-term incidence rates of serious infection and malignancy were quite low, in particular the incidence rate of malignancy (less than 1/100PYs). Despite the enhanced short-term risks of overall infection and some specific infections with both IL-17 inhibitors and IL-23 inhibitors, the risk ratios were moderately increased. The long-term incidence rates of overall infection were estimated to be 57.78/100PYs with IL-17 inhibitors and 48.5/100PYs with IL-23 inhibitors.

Subgroup analyses by biological agents suggested that brodalumab, ixekizumab, secukinumab, bimekizumab, guselkumab, risankizumab and tildrakizumab are safe in general, but still showed diversity in safety profile. In this study, ixekizumab, secukinumab, bimekizumab and risankizumab increased the short-term risk of overall infection, whereas other drugs did not. Specifically, ixekizumab might increase the short-term risk of *Candida* infection; secukinumab might increase the short-term risk of nasopharyngitis and upper respiratory tract infection; bimekizumab might increase the short-term risk of nasopharyngitis and *Candida* infection. None of guselkumab, risankizumab and tildrakizumab was able to increase the short-term risk of nasopharyngitis or upper respiratory tract infection. In general, the risks of infection and malignancy were comparable between patients with PsO and PsA, though there were some differences in individual AEs.

Nasopharyngitis and upper respiratory tract infection are considered the most frequent AEs with IL-17 or IL-23 antagonists (10, 11). It is interesting to note that IL-17 inhibitors did not increase the short-term risk of upper respiratory tract infection, and slightly increased the risk of nasopharyngitis. IL-23 inhibitors did not increase the short-term risk of nasopharyngitis or upper respiratory tract infection. But the long-term EAIRs of nasopharyngitis (15.07/100PYs with IL-17 inhibitors; 10.75/100PYs with IL-23 inhibitors) and upper respiratory tract infection (8.52/100PYs with IL-17 inhibitors; 5.84/100PYs with IL-23 inhibitors) were somewhat high. It is possible that nasopharyngitis and upper respiratory tract infection, albeit as two common infections in real-life settings with high incidence, are likely to have comparable incidence rates in psoriasis patients with or without using IL-17/IL-23 inhibitors. However, this finding requires further validation.

It is noteworthy that *Candida* infection appeared to be a prominent AE for IL-17 inhibitors. Our study suggested that the short-term risk of *Candida* infection was approximately 3-fold higher when administrating IL-17 antagonists compared with placebo. The long-term incidence rate was estimated to be 3.41/100PYs. It is well-acknowledged that IL-17 plays a role in host defense against bacterial and fungal infections, particularly in mucocutaneous microbial surveillance (86, 88). The deficiency of IL-17 immunity could lead to infections with *Candida species (* 89). Hence, continued vigilance for such infection will be required during treatment with inhibitors of this pathway. In addition, It is worth noting that the risk of *Candida* infection was increased almost 6-fold when bimekizumab was used, which appeared to be higher than other IL-17 inhibitors. These findings were consistent with data from existing clinical trials, which demonstrated a higher incidence of *Candida* infection with bimekizumab than other IL-17 antagonists (41–45, 75, 76). It might be explained by the differing targets between bimekizumab and other IL-17 inhibitors (6). In comparison to ixekizumab and secukinumab which target IL-17A, bimekizumab has been shown to have dual neutralization of IL-17A and IL-17F. These findings point to the important role of IL-17F in host defense, especially at the mucocutaneous sites (86, 88). Despite higher *Candida* infection occurrence with IL-17 antagonists, most reported cases were mild or moderate infections, none of them were systemic, and most cases could be resolved with appropriate antifungal treatment without causing trial dropouts (41–45).

Compared with *Candida* infection, the incidence rates of tuberculosis, hepatitis, and herpes zoster were quite lower. For IL-17 inhibitors, zero case of tuberculosis in the induction periods was found, and only 3 cases of latent tuberculosis in long-term extension periods were reported. For IL-23 inhibitors, no case of tuberculosis in the whole induction periods and extension periods was reported. No case of active or reactivated tuberculosis was ever reported in all the included studies and throughout the whole trial periods, either with IL-17 inhibitors or IL-23 inhibitors. Short-term risks of hepatitis and herpes zoster seemed not increased according to the meta-analyses, despite limited studies and small sample sizes. Only several cases of hepatitis and herpes zoster were reported throughout the whole trial periods. The trial researchers tended not to attribute these cases to treatment-related reasons (17, 64–66, 80, 83, 84). These results supported that IL-17 inhibitors and IL-23 inhibitors may not increase the risk of tuberculosis, hepatitis, and herpes zoster in psoriasis patients without known diagnoses or previous histories of these infections. However, it did not mean that patients with history or evidence were totally safe to receive IL-17 or IL-23 inhibitors, as patients at high risk or with previous histories of tuberculosis, hepatitis or herpes zoster were already excluded in most studies. Therefore, it is noteworthy that patients with susceptibility to opportunistic infections and hepatitis always need overall examination and prudent evaluation before and during using any biologics (6, 10).

The long-term safety analyses presented results of continuous treatments of IL-17 inhibitors and IL-23 inhibitors for up to 6 years, with > 42,000 PYs of total exposure. The cumulative long-term EAIRs of serious infection and malignancies are generally comparable to the results from the psoriasis reference population captured in the Psoriasis Longitudinal Assessment and Registry (PSOLAR) (90, 91). The long-term EAIRs of overall infection, serious infection, NMSC and malignancies excluding NMSC were also comparable between IL-17 antagonists and IL-23 antagonists. IL-23 maintains IL-17–producing Th17 cells, and is essential for their differentiation, activation, and survival (8). Therefore, inhibition of IL-23 blocks downstream production of IL-17A by Th17 cells and other cell types (87). This might explain the comparable safety profiles between IL-17 inhibitors and IL-23 inhibitors in our study. The EAIRs of infection and malignancy were stratified by years to estimate the EAIRs in different durations. While the present study was not designed to quantify the differences of EAIRs between different durations, these findings seemed to suggest that the EAIRs of infection and malignancy might be broadly consistent or even diminished with the prolonged treatment duration. These findings indicated that long-term sustained treatments with IL-17 or IL-23 inhibitors with appropriate doses could be safe with continued follow-ups of the patients.

This study has several limitations. Firstly, we did not assess the dose-related trends in the incidence rate or severity of infection and malignancy. Patients are included in our analysis once they have received at least one dose of either the anti-IL-17 or anti-IL-23 antagonists. Secondly, this study might fail to evaluate the risk in patients at high risk for infections or malignancies because of the possible exclusion of patients with previous histories of infections or malignancies in the original clinical studies. Thirdly, some of the results of the meta-analyses may still present small sample sizes due to the limited data, which may decrease the reliability. Finally, we only included RCTs and OLE studies in this review. There is a need for a meta-analysis of real-world studies evaluating the risk of infection and malignancy in patients with psoriatic disease who receive IL-17 or IL-23 antagonists.

In conclusion, IL-17 inhibitors and IL-23 inhibitors are generally safe in the treatment of psoriasis and psoriatic arthritis, with both short-term and long-term treatments. Nasopharyngitis and upper respiratory tract infection are the most frequent AEs, and *Candida* infection seemed to be the most common opportunistic infection. It is critical to exercise caution and maintain ongoing vigilance for nasopharyngitis and *Candida* infection in patients receiving IL-17 inhibitors. However, to comprehensively evaluate the potential risks of cancer and infection related to prolonged use of IL-17 inhibitors and IL-23 inhibitors in individuals with psoriasis, larger, long-term, prospective cohort studies will be required.

## Data availability statement

The original contributions presented in the study are included in the article/**Supplementary Material**. Further inquiries can be directed to the corresponding author.

## Author contributions

SW: Conceptualization, Data curation, Formal Analysis, Methodology, Writing – original draft, Writing – review & editing, Investigation, Software, Visualization. YX: Formal Analysis, Methodology, Software, Writing – original draft, Writing – review & editing, Data curation. LY: Formal Analysis, Methodology, Writing – original draft, Writing – review & editing, Investigation, Software. LG: Funding acquisition, Supervision, Validation, Writing – original draft, Writing – review & editing, Investigation, Methodology, Visualization. XJ: Funding acquisition, Supervision, Validation, Writing – original draft, Writing – review & editing, Conceptualization, Methodology, Resources.
