# Supplementary material for: Short-term risk and long-term incidence rate of infection and malignancy with IL-17 and IL-23 inhibitors in adult patients with psoriasis and psoriatic arthritis: a systematic review and meta-analysis
Source: Front Immunol. 2023 Nov 29;14:1294416. doi: 10.3389/fimmu.2023.1294416 (PMC10721978; doi:10.3389/fimmu.2023.1294416)
Supplement: Supplementary file 1 [file DataSheet_1.docx]

Supplementary Material

# Supplementary Tables

**Table S1.** A completed PRISMA (Preferred Reporting Item for Systematic Reviews and Meta-Analyses) checklist

| **Section and Topic** | **Item #** | **Checklist item** | **Location where item is reported** |
| --- | --- | --- | --- |
| **TITLE** | | |  |
| Title | 1 | Identify the report as a systematic review. | ✓ |
| **ABSTRACT** | | |  |
| Abstract | 2 | See the PRISMA 2020 for Abstracts checklist. | ✓ |
| **INTRODUCTION** | | |  |
| Rationale | 3 | Describe the rationale for the review in the context of existing knowledge. | ✓ |
| Objectives | 4 | Provide an explicit statement of the objective(s) or question(s) the review addresses. | ✓ |
| **METHODS** | | |  |
| Eligibility criteria | 5 | Specify the inclusion and exclusion criteria for the review and how studies were grouped for the syntheses. | ✓ |
| Information sources | 6 | Specify all databases, registers, websites, organisations, reference lists and other sources searched or consulted to identify studies. Specify the date when each source was last searched or consulted. | ✓ |
| Search strategy | 7 | Present the full search strategies for all databases, registers and websites, including any filters and limits used. | ✓ |
| Selection process | 8 | Specify the methods used to decide whether a study met the inclusion criteria of the review, including how many reviewers screened each record and each report retrieved, whether they worked independently, and if applicable, details of automation tools used in the process. | ✓ |
| Data collection process | 9 | Specify the methods used to collect data from reports, including how many reviewers collected data from each report, whether they worked independently, any processes for obtaining or confirming data from study investigators, and if applicable, details of automation tools used in the process. | ✓ |
| Data items | 10a | List and define all outcomes for which data were sought. Specify whether all results that were compatible with each outcome domain in each study were sought (e.g. for all measures, time points, analyses), and if not, the methods used to decide which results to collect. | ✓ |
|  | 10b | List and define all other variables for which data were sought (e.g. participant and intervention characteristics, funding sources). Describe any assumptions made about any missing or unclear information. | ✓ |
| Study risk of bias assessment | 11 | Specify the methods used to assess risk of bias in the included studies, including details of the tool(s) used, how many reviewers assessed each study and whether they worked independently, and if applicable, details of automation tools used in the process. | ✓ |
| Effect measures | 12 | Specify for each outcome the effect measure(s) (e.g. risk ratio, mean difference) used in the synthesis or presentation of results. | ✓ |
| Synthesis methods | 13a | Describe the processes used to decide which studies were eligible for each synthesis (e.g. tabulating the study intervention characteristics and comparing against the planned groups for each synthesis (item #5)). | ✓ |
|  | 13b | Describe any methods required to prepare the data for presentation or synthesis, such as handling of missing summary statistics, or data conversions. | ✓ |
|  | 13c | Describe any methods used to tabulate or visually display results of individual studies and syntheses. | ✓ |
|  | 13d | Describe any methods used to synthesize results and provide a rationale for the choice(s). If meta-analysis was performed, describe the model(s), method(s) to identify the presence and extent of statistical heterogeneity, and software package(s) used. | ✓ |
|  | 13e | Describe any methods used to explore possible causes of heterogeneity among study results (e.g. subgroup analysis, meta-regression). | ✓ |
|  | 13f | Describe any sensitivity analyses conducted to assess robustness of the synthesized results. | ✓ |
| Reporting bias assessment | 14 | Describe any methods used to assess risk of bias due to missing results in a synthesis (arising from reporting biases). | None, ITT was used |
| Certainty assessment | 15 | Describe any methods used to assess certainty (or confidence) in the body of evidence for an outcome. | ✓ |
| **RESULTS** | | |  |
| Study selection | 16a | Describe the results of the search and selection process, from the number of records identified in the search to the number of studies included in the review, ideally using a flow diagram. | ✓ |
|  | 16b | Cite studies that might appear to meet the inclusion criteria, but which were excluded, and explain why they were excluded. | Not cited due to too many studies |
| Study characteristics | 17 | Cite each included study and present its characteristics. | ✓ |
| Risk of bias in studies | 18 | Present assessments of risk of bias for each included study. | ✓ |
| Results of individual studies | 19 | For all outcomes, present, for each study: (a) summary statistics for each group (where appropriate) and (b) an effect estimate and its precision (e.g. confidence/credible interval), ideally using structured tables or plots. | ✓ |
| Results of syntheses | 20a | For each synthesis, briefly summarise the characteristics and risk of bias among contributing studies. | ✓ |
|  | 20b | Present results of all statistical syntheses conducted. If meta-analysis was done, present for each the summary estimate and its precision (e.g. confidence/credible interval) and measures of statistical heterogeneity. If comparing groups, describe the direction of the effect. | ✓ |
|  | 20c | Present results of all investigations of possible causes of heterogeneity among study results. | ✓ |
|  | 20d | Present results of all sensitivity analyses conducted to assess the robustness of the synthesized results. | ✓ |
| Reporting biases | 21 | ✓ | ✓ |
| Certainty of evidence | 22 | Present assessments of certainty (or confidence) in the body of evidence for each outcome assessed. | ✓ |
| **DISCUSSION** | | |  |
| Discussion | 23a | Provide a general interpretation of the results in the context of other evidence. | ✓ |
|  | 23b | Discuss any limitations of the evidence included in the review. | ✓ |
|  | 23c | Discuss any limitations of the review processes used. | ✓ |
|  | 23d | Discuss implications of the results for practice, policy, and future research. | ✓ |
| **OTHER INFORMATION** | | |  |
| Registration and protocol | 24a | Provide registration information for the review, including register name and registration number, or state that the review was not registered. | ✓ |
|  | 24b | Indicate where the review protocol can be accessed, or state that a protocol was not prepared. | ✓ |
|  | 24c | Describe and explain any amendments to information provided at registration or in the protocol. | No amendments were made |
| Support | 25 | Describe sources of financial or non-financial support for the review, and the role of the funders or sponsors in the review. | ✓ |
| Competing interests | 26 | Declare any competing interests of review authors. | ✓ |
| Availability of data, code and other materials | 27 | Report which of the following are publicly available and where they can be found: template data collection forms; data extracted from included studies; data used for all analyses; analytic code; any other materials used in the review. | ✓ |

*From:*  Page MJ, McKenzie JE, Bossuyt PM, Boutron I, Hoffmann TC, Mulrow CD, et al. The PRISMA 2020 statement: an updated guideline for reporting systematic reviews. BMJ 2021;372:n71. doi: 10.1136/bmj.n71

**Table S2.** Original Data of Primary Outcomes Extracted from Included Placebo-controlled RCTs.

| **Source** | **Trial Name/ ClinicalTrials.gov identifier** | **Treatment (dose)** | **Treatment Group** | | | | |  | **Placebo Group** | | | | |
| --- | --- | --- | --- | --- | --- | --- | --- | --- | --- | --- | --- | --- | --- |
|  |  |  | **Number of Patients** | **Patients with Serious Infection** | **Patients with Infection** | **Patients with Malignancy** | **Type of Malignancy** |  | **Number of Patients** | **Patients with Serious Infection** | **Patients with Infection** | **Patients with Malignancy** | **Type of Malignancy** |
| Papp *et al*, 2016 | AMAGINE-1/ NCT01708590 | Brodalumab (140 mg, Q2W)  Brodalumab (210 mg, Q2W) | 219  222 | 2  1 | NA  NA | NA  NA | NA  NA |  | 220 | 0 | NA | NA | NA |
| Lebwohl *et al*, 2015 | AMAGINE-2/ NCT01708603 | Brodalumab (140 mg, Q2W)  Brodalumab (210 mg, Q2W) | 610  612 | 3  2 | 130  148 | 0  0 | NA |  | 309 | 2 | 58 | 0 | NA |
| Lebwohl *et al*, 2015 | AMAGINE-3/ NCT01708629 | Brodalumab (140 mg, Q2W)  Brodalumab (210 mg, Q2W) | 629  624 | 1  1 | 70  92 | 0  0 | NA |  | 315 | 1 | 50 | 0 | NA |
| Mease *et al*, 2021 | AMVISION-1/ NCT02029495;  AMVISION-2/ NCT02024646 | Brodalumab (140 mg, at weeks 0, 1, then Q2W)  Brodalumab (210 mg, at weeks 0, 1, then Q2W) | 318  321 | 0  1 | 75  96 | 1  1 | Malignant Melanoma;  Squamous Cell Carcinoma of the Vulva |  | 320 | 0 | 91 | 0 | NA |
| Seo *et al*,  2021 | NCT02982005 | Brodalumab (210 mg, at weeks 0, 1, then Q2W) | 40 | NA | NA | 0 | NA |  | 22 | NA | NA | 0 | NA |
| Mease *et al*, 2014 | NCT01516957 | Brodalumab (140 mg, at weeks 0, 1, then Q2W)  Brodalumab (280 mg, at weeks 0, 1, then Q2W) | 56  56 | 0  1 | NA  NA | 1  2 | Breast Cancer;  Lung Cancer Metastatic; Malignant Melinoma |  | 55 | 1 | NA | 1 | Breast Cancer |
| Papp *et al*, 2012 | NCT00975637 | Brodalumab (70 mg, at weeks 0, 1, then Q2W)  Brodalumab (140 mg, at weeks 0, 1, then Q2W)  Brodalumab (210 mg, at weeks 0, 1, then Q2W)  Brodalumab (280 mg, monthly) | 39  39  40  41 | NA  NA  NA  NA | NA  NA  NA  NA | 0  0  0  0 | NA |  | 38 | NA | NA | 0 | NA |
| Nakagawa *et al*, 2016 | NCT01748539 | Brodalumab (70 mg, at weeks 0, 1, then Q2W)  Brodalumab (140 mg, at weeks 0, 1, then Q2W)  Brodalumab (210 mg, at weeks 0, 1, then Q2W) | 39  37  37 | NA  NA  NA | NA  NA  NA | NA  NA  NA | NA  NA  NA |  | 38 | NA | NA | NA | NA |
| Gordon *et al*, 2016 | UNCOVER-1/ NCT01474512;  UNCOVER-2/ NCT01597245;  UNCOVER-3/  NCT01646177 | Ixekizumab (160 mg at week 0, then 80 mg Q2W)  Ixekizumab (160 mg at week 0, then 80 mg Q4W) | 1167  1161 | 5  8 | 315  318 | 3  3 | 1*NMSC, 2*other malignancies;  2*NMSC, 1*other malignancy |  | 791 | 3 | 181 | 2 | 1*NMSC, 1*other malignancy |
| Mease *et al*, 2017 | SPIRIT-P1/ NCT01695239 | Ixekizumab (160 mg at week 0, then 80 mg Q2W)  Ixekizumab (160 mg at week 0, then 80 mg Q4W) | 102  107 | 2  1 | 24  30 | 0  0 | NA  NA |  | 106 | 0 | 27 | 1 | Unknown |
| Nash *et al*, 2017 | SPIRIT-P2/ NCT02349295 | Ixekizumab (160 mg at week 0, then 80 mg Q2W)  Ixekizumab (160 mg at week 0, then 80 mg Q4W) | 123  122 | 3  0 | 47  47 | 0  0 | NA  NA |  | 118 | 0 | 35 | 0 | NA |
| Ryan *et al*, 2018 | IXORA-Q/ NCT02718898 | Ixekizumab (160 mg at week 0, then 80 mg Q2W) | 75 | 0 | 14 | 0 | NA |  | 74 | 0 | 9 | 0 | NA |
| Leonardi *et al*, 2012 | NCT01107457 | Ixekizumab (10 mg, at weeks 0, 2, 4, 8, 12, and 16)  Ixekizumab (25 mg, at weeks 0, 2, 4, 8, 12, and 16)  Ixekizumab (75 mg, at weeks 0, 2, 4, 8, 12, and 16)  Ixekizumab (150 mg, at weeks 0, 2, 4, 8, 12, and 16) | 28  30  29  28 | NA  NA  NA  NA | 12  9  9  8 | 0  0  0  0 | NA  NA  NA  NA |  | 27 | NA | 7 | 0 | NA |
| Langley *et al*, 2014 | ERASURE/ NCT01365455 | Secukinumab (150 mg, QW for 5 weeks, then Q4W)  Secukinumab (300 mg, QW for 5 weeks, then Q4W) | 245  245 | NA  NA | 66  72 | NA  NA | NA  NA |  | 247 | NA | 40 | NA | NA |
| Langley *et al*, 2014 | FIXTURE/ NCT01358578 | Secukinumab (150 mg, QW for 5 weeks, then Q4W)  Secukinumab (300 mg, QW for 5 weeks, then Q4W) | 327  326 | NA  NA | 101  87 | NA  NA | NA  NA |  | 327 | NA | 63 | NA | NA |
| Mease *et al*, 2015 | FUTURE1/ NCT01392326 | Secukinumab (10 mg per kilogram at weeks 0, 2, and 4, then 75 mg Q4W)  Secukinumab (10 mg per kilogram at weeks 0, 2, and 4, then 150 mg Q4W) | 202  202 | NA  NA | 53  67 | NA  NA | NA  NA |  | 202 | NA | 47 | NA | NA |
| McInnes *et al*, 2015 | FUTURE2/ NCT01752634 | Secukinumab (75 mg, QW for 4 weeks, then Q4W)  Secukinumab (150 mg, QW for 4 weeks, then Q4W)  Secukinumab (300 mg, QW for 4 weeks, then Q4W) | 99  100  100 | NA  NA  NA | 23  30  29 | NA  NA  NA | NA  NA  NA |  | 98 | NA | 30 | NA | NA |
| Nash *et al*, 2018 | FUTURE3/ NCT01989468 | Secukinumab (150 mg, QW for 4 weeks, then Q4W)  Secukinumab (300 mg, QW for 4 weeks, then Q4W) | 138  139 | NA  NA | NA  NA | NA  NA | NA  NA |  | 137 | NA | NA | NA | NA |
| Mease *et al*, 2018 | FUTURE5/ NCT02404350 | Secukinumab (150 mg with loading dose, QW for 4 weeks, then Q4W)  Secukinumab (150 mg without loading dose, QW for 4 weeks, then Q4W)  Secukinumab (300 mg, QW for 4 weeks, then Q4W) | 822 | NA | NA | 1 | Melanoma |  | 332 | NA | NA | 0 | NA |
| Baraliakos *et al*, 2021 | MAXIMISE/ NCT02721966 | Secukinumab (150 mg, QW for 4 weeks, then Q4W)  Secukinumab (300 mg, QW for 4 weeks, then Q4W) | 165  167 | 1  6 | 95  103 | 0  0 | NA  NA |  | 166 | 1 | 27 | 0 | NA |
| Gottlieb *et al*, 2017 | GESTURE/ NCT01806597 | Secukinumab (150 mg, QW for 4 weeks, then Q4W)  Secukinumab (300 mg, QW for 4 weeks, then Q4W) | 68  69 | NA  NA | NA  NA | NA  NA | NA  NA |  | 68 | NA | NA | NA | NA |
| Paul *et al*, 2015 | JUNCTURE/  NCT01636687 | Secukinumab (150 mg, QW for 4 weeks, then Q4W)  Secukinumab (300 mg, QW for 4 weeks, then Q4W) | 61  60 | 1  0 | NA  NA | 1  0 | melanoma in situ;  NA |  | 61 | 0 | NA | 0 | NA |
| Bagel *et al*, 2017 | NCT02267135 | Secukinumab (300 mg, QW for 4 weeks, then Q4W) | 51 | 0 | 15 | NA | NA |  | 51 | 1 | 10 | NA | NA |
| McInnes *et al*, 2014 | NCT00809614 | Secukinumab (10mg/kg, on day 1 and day 22) | 28 | NA | 23 | NA | NA |  | 14 | NA | 7 | NA | NA |
| Nguyen *et al*, 2022 | CHOICE/  NCT02798211 | Secukinumab (150 mg, QW for 4 weeks, then Q4W)  Secukinumab (300 mg, QW for 4 weeks, then Q4W) | 103  103 | NA  NA | NA  NA | NA  NA | NA  NA |  | 52 | NA | NA | NA | NA |
| Papp *et al*, 2013 | NCT01071252 | Secukinumab (1*25 mg, at weeks 0, 4 and 8)  Secukinumab (3*25 mg, at weeks 0, 4 and 8)  Secukinumab (3*75 mg, at weeks 0, 4 and 8)  Secukinumab (3*150 mg, at weeks 0, 4 and 8) | 103 | 1 | NA | NA | NA |  | 22 | 0 | NA | NA | NA |
| Rich *et al*, 2013 | NCT00941031 | Secukinumab (150 mg, at week 0)  Secukinumab (150 mg, at weeks 0, 4 and 8)  Secukinumab (150 mg, at weeks 0, 1, 2 and 4) | 66  138  133 | NA  NA  NA | 14  56  45 | NA  NA  NA | NA  NA  NA |  | 67 | NA | 26 | NA | NA |
| Gordon *et al*, 2021 | BE READY/  NCT03410992 | Bimekizumab (320 mg Q4W) | 349 | 2 | NA | 2 | NMSC; prostate cancer |  | 86 | 0 | NA | 0 | NA |
| McInne *et al*, 2023 | BE OPTIMAL/ NCT03895203 | Bimekizumab (160 mg Q4W) | 431 | 1 | 131 | 1 | NMSC |  | 281 | 0 | 56 | 1 | Breast cancer |
| Merola *et al*, 2023 | BE COMPLETE/  NCT03896581 | Bimekizumab (160 mg Q4W) | 267 | 2 | NA | 0 | NA |  | 132 | 0 | NA | 1 | NMSC |
| Reich *et al*, 2021 | BE VIVID/  NCT03370133 | Bimekizumab (320 mg Q4W) | 321 | 0 | NA | 0 | NA |  | 83 | 0 | NA | 1 | Oesophageal adenocarcinoma |
| Ritchlin *et al*, 2020 | BE ACTIVE/ NCT02969525 | Bimekizumab (16 mg Q4W);  Bimekizumab, (160 mg Q4W);  Bimekizumab, (160 mg Q4W, loading  dose);  Bimekizumab (320 mg Q4W) | 39  43  41  41 | 0  0  2  0 | NA  NA  NA  NA | 0  0  0  0 | NA  NA  NA  NA |  | 42 | 0 | NA | 0 | NA |
| Blauvelt *et al*, 2017 | VOYAGE 1/ NCT02207231 | Guselkumab (100 mg, at weeks 0 and 4, then Q8W) | 329 | 0 | 85 | 1 | NMSC |  | 174 | 0 | 44 | 0 | NA |
| Reich *et al*, 2017 | VOYAGE 2/ NCT02207244 | Guselkumab (100 mg, at weeks 0 and 4, then Q8W) | 494 | 1 | 106 | 0 | NA |  | 248 | 1 | 46 | 0 | NA |
| Coates *et al*, 2022 | COSMOS/  NCT03796858 | Guselkumab (100 mg, at weeks 0 and 4, then Q8W) | 189 | 1 | 40 | 1 | Prostate cancer |  | 96 | 0 | 19 | 0 | NA |
| Deodhar *et al*, 2020 | DISCOVER-1/  NCT03162796 | Guselkumab (100 mg, Q4W)  Guselkumab (100 mg, at weeks 0 and 4, then Q8W) | 128  127 | 0  0 | 31  33 | 0  1 | NA;  Plasma cell myeloma |  | 126 | 2 | 32 | 0 | NA |
| Mease *et al*, 2020 | DISCOVER-2/  NCT03158285 | Guselkumab (100 mg, Q4W)  Guselkumab (100 mg, at weeks 0 and 4, then Q8W) | 245  248 | 3  1 | 49  40 | 0  1 | NA;  Melanoma in situ |  | 246 | 1 | 45 | 1 | Clear-cell renal cell carcinoma |
| Ohtsuki *et al*, 2018 | NCT02325219 | Guselkumab (50 mg, at weeks 0 and 4, then Q8W)  Guselkumab (100 mg, at weeks 0 and 4, then Q8W) | 65  63 | 0  1 | 18  15 | 1  0 | Rectal adenocarcinoma;  NA |  | 64 | 1 | 14 | 0 | NA |
| Deodhar *et al*, 2018 | NCT02319759 | Guselkumab (100 mg, at weeks 0 and 4, then Q8W) | 100 | 0 | 16 | NA | NA |  | 49 | 0 | 10 | NA | NA |
| Gordon *et al*, 2015 | NCT01483599 | Guselkumab (5 mg, at weeks 0 and 4, then Q12W)  Guselkumab (15 mg Q8W)  Guselkumab (50 mg, at weeks 0 and 4, then Q12W)  Guselkumab (100 mg Q8W)  Guselkumab (200 mg, at weeks 0 and 4, then Q12W) | 207 | 2 | 41 | NA | NA |  | 42 | 0 | 6 | NA | NA |
| Gordon *et al*, 2018 | UltIMMa-1/  NCT02684370 | Risankizumab (150 mg, at weeks 0, 4, and 16) | 304 | 1 | 75 | 1 | Squamous cell carcinoma |  | 102 | 0 | 17 | 1 | Squamous cell carcinoma |
| Gordon *et al*, 2018 | UltIMMa-2/  NCT02684357 | Risankizumab (150 mg, at weeks 0, 4, and 16) | 294 | 3 | 56 | 1 | Basal cell carcinoma |  | 98 | 0 | 9 | 0 | NA |
| Kristensen *et al*, 2022 | KEEPsAKE 1/  NCT03675308 | Risankizumab (150 mg, at weeks 0, 4, and 16) | 483 | 5 | NA | 0 | NA |  | 481 | 6 | NA | 2 | Breast cancer;  Non-small-cell lung cancer |
| Östör *et al*, 2022 | KEEPsAKE 2/  NCT03671148 | Risankizumab (150 mg, at weeks 0, 4, and 16) | 224 | 2 | NA | 1 | NMSC |  | 219 | 5 | NA | 1 | NMSC |
| Blauvelt *et al*, 2020 | NCT02672852 | Risankizumab (150 mg, at weeks 0, 4, and 16) | 407 | 1 | 70 | 3 | Esophageal carcinoma; Malignant melanoma in situ; Squamous cell carcinoma in situ |  | 100 | 1 | 18 | 0 | NA |
| Reich *et al*, 2017 | reSURFACE 1/  NCT01722331 | Tildrakizumab (100 mg, at weeks 0, 4, and 16)  Tildrakizumab (200 mg, at weeks 0, 4, and 16) | 309  308 | 1  1 | 34  35 | 0  0 | NA  NA |  | 154 | 0 | 17 | 0 | NA |
| Reich *et al*, 2017 | reSURFACE 2/  NCT01729754 | Tildrakizumab (100 mg, at weeks 0, 4, and 16)  Tildrakizumab (200 mg, at weeks 0, 4, and 16) | 307  314 | 0  1 | 41  35 | 1  1 | NMSC;  NMSC |  | 156 | 1 | 12 | 0 | NA |
| Papp *et al*, 2015 | NCT01225731 | Tildrakizumab (5 mg, at weeks 0, 4, and 16)  Tildrakizumab (25 mg, at weeks 0, 4, and 16)  Tildrakizumab (100 mg, at weeks 0, 4, and 16)  Tildrakizumab (200 mg, at weeks 0, 4, and 16) | 42  91  89  86 | 0  0  1  0 | NA  NA  NA  NA | 0  0  0  0 | NA  NA  NA  NA |  | 45 | 0 | NA | 0 | NA |

NA, not available; NMSC, nonmelanoma skin cancer; QW, every week; Q4W, every 4 weeks; Q8W, every 8 weeks; Q12W, every 12 weeks.

**Table S3.** Original Data of Secondary Outcomes Extracted from Included Placebo-controlled RCTs

| **Source** | **Trial Name/ ClinicalTrials.gov identifier** | **Treatment (dose)** | **Treatment Group** | | | |  | **Placebo Group** | | | |
| --- | --- | --- | --- | --- | --- | --- | --- | --- | --- | --- | --- |
|  |  |  | **Number of Patients** | **Patients with Nasophar-yngitis** | **Patients with Upper Respiratory Tract Infection** | **Patients with Candida Infection** |  | **Number of Patients** | **Patients with Nasopharyngitis** | **Patients with Upper Respiratory Tract Infection** | **Patients with Candida Infection** |
| Papp *et al*, 2016 | AMAGINE-1/ NCT01708590 | Brodalumab (140 mg, Q2W)  Brodalumab (210 mg, Q2W) | 219  222 | 20  21 | 18  18 | 1  5 |  | 220 | 22 | 14 | 3 |
| Lebwohl *et al*, 2015 | AMAGINE-2/ NCT01708603 | Brodalumab (140 mg, Q2W)  Brodalumab (210 mg, Q2W) | 610  612 | NA | NA | NA |  | 309 | NA | NA | NA |
| Lebwohl *et al*, 2015 | AMAGINE-3/ NCT01708629 | Brodalumab (140 mg, Q2W)  Brodalumab (210 mg, Q2W) | 629  624 | NA | NA | NA |  | 315 | NA | NA | NA |
| Mease *et al*, 2021 | AMVISION-1/ NCT02029495;  AMVISION-2/ NCT02024646 | Brodalumab (140 mg, at weeks 0, 1, then Q2W)  Brodalumab (210 mg, at weeks 0, 1, then Q2W) | 318  321 | NA  NA | NA  NA | NA  NA |  | 320 | NA | NA | NA |
| Seo *et al*,  2021 | NCT02982005 | Brodalumab (210 mg, at weeks 0, 1, then Q2W) | 40 | 1 | 1 | NA |  | 22 | 0 | 2 | 0 |
| Mease *et al*, 2014 | NCT01516957 | Brodalumab (140 mg, at weeks 0, 1, then Q2W)  Brodalumab (280 mg, at weeks 0, 1, then Q2W) | 56  56 | 0  0 | 5  8 | NA  NA |  | 55 | 3 | 4 | NA |
| Papp *et al*, 2012 | NCT00975637 | Brodalumab (70 mg, at weeks 0, 1, then Q2W)  Brodalumab (140 mg, at weeks 0, 1, then Q2W)  Brodalumab (210 mg, at weeks 0, 1, then Q2W)  Brodalumab (280 mg, monthly) | 39  39  40  41 | 6  1  4  2 | 3  3  2  5 | NA  NA  NA  NA |  | 38 | 3 | 2 | NA |
| Nakagawa *et al*, 2016 | NCT01748539 | Brodalumab (70 mg, at weeks 0, 1, then Q2W)  Brodalumab (140 mg, at weeks 0, 1, then Q2W)  Brodalumab (210 mg, at weeks 0, 1, then Q2W) | 39  37  37 | 5  5  4 | 2  2  0 | NA  NA  NA |  | 38 | 3 | 0 | NA |
| Gordon *et al*, 2016 | UNCOVER-1/ NCT01474512;  UNCOVER-2/ NCT01597245;  UNCOVER-3/  NCT01646177 | Ixekizumab (160 mg at week 0, then 80 mg Q2W)  Ixekizumab (160 mg at week 0, then 80 mg Q4W) | 1167  1161 | 111  104 | 51  45 | 16  7 |  | 791 | 69 | 28 | 4 |
| Mease *et al*, 2017 | SPIRIT-P1/ NCT01695239 | Ixekizumab (160 mg at week 0, then 80 mg Q2W)  Ixekizumab (160 mg at week 0, then 80 mg Q4W) | 102  107 | 3  7 | 3  5 | 1  1 |  | 106 | 5 | 7 | 0 |
| Nash *et al*, 2017 | SPIRIT-P2/ NCT02349295 | Ixekizumab (160 mg at week 0, then 80 mg Q2W)  Ixekizumab (160 mg at week 0, then 80 mg Q4W) | 123  122 | 4  8 | 12  11 | 6  2 |  | 118 | 4 | 9 | 0 |
| Ryan *et al*, 2018 | IXORA-Q/ NCT02718898 | Ixekizumab (160 mg at week 0, then 80 mg Q2W) | 75 | NA | NA | NA |  | 74 | NA | NA | NA |
| Leonardi *et al*, 2012 | NCT01107457 | Ixekizumab (10 mg, at weeks 0, 2, 4, 8, 12, and 16)  Ixekizumab (25 mg, at weeks 0, 2, 4, 8, 12, and 16)  Ixekizumab (75 mg, at weeks 0, 2, 4, 8, 12, and 16)  Ixekizumab (150 mg, at weeks 0, 2, 4, 8, 12, and 16) | 28  30  29  28 | 3  3  3  4 | 1  3  1  1 | NA  NA  NA  NA |  | 27 | 5 | 1 | NA |
| Langley *et al*, 2014 | ERASURE/ NCT01365455 | Secukinumab (150 mg, QW for 5 weeks, then Q4W)  Secukinumab (300 mg, QW for 5 weeks, then Q4W) | 245  245 | 23  22 | 10  9 | NA  NA |  | 247 | 19 | 0 | NA |
| Langley *et al*, 2014 | FIXTURE/ NCT01358578 | Secukinumab (150 mg, QW for 5 weeks, then Q4W)  Secukinumab (300 mg, QW for 5 weeks, then Q4W) | 327  326 | 45  35 | 10  7 | NA  NA |  | 327 | 26 | 3 | NA |
| Mease *et al*, 2015 | FUTURE1/ NCT01392326 | Secukinumab (10 mg per kilogram at weeks 0, 2, and 4, then 75 mg Q4W)  Secukinumab (10 mg per kilogram at weeks 0, 2, and 4, then 150 mg Q4W) | 202  202 | 14  19 | 9  13 | 1  2 |  | 202 | 9 | 10 | 0 |
| McInnes *et al*, 2015 | FUTURE2/ NCT01752634 | Secukinumab (75 mg, QW for 4 weeks, then Q4W)  Secukinumab (150 mg, QW for 4 weeks, then Q4W)  Secukinumab (300 mg, QW for 4 weeks, then Q4W) | 99  100  100 | 6  4  6 | 10  8  4 | NA  NA  NA |  | 98 | 8 | 7 | NA |
| Nash *et al*, 2018 | FUTURE3/ NCT01989468 | Secukinumab (150 mg, QW for 4 weeks, then Q4W)  Secukinumab (300 mg, QW for 4 weeks, then Q4W) | 138  139 | 11  13 | 6  7 | 2  0 |  | 137 | 13 | 15 | 0 |
| Mease *et al*, 2018 | FUTURE5/ NCT02404350 | Secukinumab (150 mg with loading dose, QW for 4 weeks, then Q4W)  Secukinumab (150 mg without loading dose, QW for 4 weeks, then Q4W)  Secukinumab (300 mg, QW for 4 weeks, then Q4W) | 822 | NA | 38 | 7 |  | 332 | NA | 11 | 2 |
| Baraliakos *et al*, 2021 | MAXIMISE/ NCT02721966 | Secukinumab (150 mg, QW for 4 weeks, then Q4W)  Secukinumab (300 mg, QW for 4 weeks, then Q4W) | 165  167 | 4  9 | NA  NA | 2  3 |  | 166 | 11 | NA | 1 |
| Gottlieb *et al*, 2017 | GESTURE/ NCT01806597 | Secukinumab (150 mg, QW for 4 weeks, then Q4W)  Secukinumab (300 mg, QW for 4 weeks, then Q4W) | 68  69 | 5  2 | 4  3 | NA  NA |  | 68 | 4 | 3 | NA |
| Paul *et al*, 2015 | JUNCTURE/  NCT01636687 | Secukinumab (150 mg, QW for 4 weeks, then Q4W)  Secukinumab (300 mg, QW for 4 weeks, then Q4W) | 61  60 | 14  19 | NA  NA | NA  NA |  | 61 | 10 | NA | NA |
| Bagel *et al*, 2017 | NCT02267135 | Secukinumab (300 mg, QW for 4 weeks, then Q4W) | 51 | 3 | 3 | NA |  | 51 | 1 | 2 | NA |
| McInnes *et al*, 2014 | NCT00809614 | Secukinumab (10mg/kg, on day 1 and day 22) | 28 | 7 | NA | NA |  | 14 | 5 | NA | NA |
| Nguyen *et al*, 2022 | CHOICE/  NCT02798211 | Secukinumab (150 mg, QW for 4 weeks, then Q4W)  Secukinumab (300 mg, QW for 4 weeks, then Q4W) | 103  103 | 4  3 | 2  6 | 2  1 |  | 52 | 1 | 0 | 0 |
| Papp *et al*, 2013 | NCT01071252 | Secukinumab (1*25 mg, at weeks 0, 4 and 8)  Secukinumab (3*25 mg, at weeks 0, 4 and 8)  Secukinumab (3*75 mg, at weeks 0, 4 and 8)  Secukinumab (3*150 mg, at weeks 0, 4 and 8) | 29  26  21  27 | 1  4  4  4 | 3  2  1  2 | NA  NA  NA  NA |  | 22 | 2 | 0 | NA |
| Rich *et al*, 2013 | NCT00941031 | Secukinumab (150 mg, at week 0)  Secukinumab (150 mg, at weeks 0, 4 and 8)  Secukinumab (150 mg, at weeks 0, 1, 2 and 4) | 66  138  133 | 8  31  30 | 3  6  2 | NA  NA  NA |  | 67 | 12 | 6 | NA |
| Gordon *et al*, 2021 | BE READY/  NCT03410992 | Bimekizumab (320 mg Q4W) | 349 | 23 | 14 | 21 |  | 86 | 4 | 7 | 0 |
| McInne *et al*, 2023 | BE OPTIMAL/ NCT03895203 | Bimekizumab (160 mg Q4W) | 431 | 40 | 21 | 11 |  | 281 | 13 | 18 | 2 |
| Merola *et al*, 2023 | BE COMPLETE/  NCT03896581 | Bimekizumab (160 mg Q4W) | 267 | 10 | 6 | 7 |  | 132 | 1 | 2 | 0 |
| Reich *et al*, 2021 | BE VIVID/  NCT03370133 | Bimekizumab (320 mg Q4W) | 321 | 30 | 9 | 28 |  | 83 | 7 | 2 | 0 |
| Ritchlin *et al*, 2020 | BE ACTIVE/ NCT02969525 | Bimekizumab (16 mg Q4W);  Bimekizumab, (160 mg Q4W);  Bimekizumab, (160 mg Q4W, loading  dose);  Bimekizumab (320 mg Q4W) | 39  43  41  41 | 3  1  1  1 | 1  0  1  1 | 0  1  2  0 |  | 42 | 0 | 0 | 0 |
| Blauvelt *et al*, 2017 | VOYAGE 1/ NCT02207231 | Guselkumab (100 mg, at weeks 0 and 4, then Q8W) | 329 | 30 | 25 | NA |  | 174 | 17 | 9 | NA |
| Reich *et al*, 2017 | VOYAGE 2/ NCT02207244 | Guselkumab (100 mg, at weeks 0 and 4, then Q8W) | 494 | 35 | 16 | NA |  | 248 | 16 | 10 | NA |
| Coates *et al*, 2022 | COSMOS/  NCT03796858 | Guselkumab (100 mg, at weeks 0 and 4, then Q8W) | 189 | 10 | 7 | NA |  | 96 | 5 | 3 | NA |
| Deodhar *et al*, 2020 | DISCOVER-1/  NCT03162796 | Guselkumab (100 mg, Q4W)  Guselkumab (100 mg, at weeks 0 and 4, then Q8W) | 128  127 | 7  16 | 11  7 | NA  NA |  | 126 | 8 | 8 | NA |
| Mease *et al*, 2020 | DISCOVER-2/  NCT03158285 | Guselkumab (100 mg, Q4W)  Guselkumab (100 mg, at weeks 0 and 4, then Q8W) | 245  248 | 12  10 | 12  6 | NA  NA |  | 246 | 9 | 8 | NA |
| Ohtsuki *et al*, 2018 | NCT02325219 | Guselkumab (50 mg, at weeks 0 and 4, then Q8W)  Guselkumab (100 mg, at weeks 0 and 4, then Q8W) | 65  63 | 14  8 | 1  2 | NA  NA |  | 64 | 7 | 1 | NA |
| Deodhar *et al*, 2018 | NCT02319759 | Guselkumab (100 mg, at weeks 0 and 4, then Q8W) | 100 | 6 | 1 | NA |  | 49 | 5 | 1 | NA |
| Gordon *et al*, 2015 | NCT01483599 | Guselkumab (5 mg, at weeks 0 and 4, then Q12W)  Guselkumab (15 mg Q8W)  Guselkumab (50 mg, at weeks 0 and 4, then Q12W)  Guselkumab (100 mg Q8W)  Guselkumab (200 mg, at weeks 0 and 4, then Q12W) | 41  41  42  42  41 | 14 | 7 | NA |  | 42 | 1 | 1 | NA |
| Gordon *et al*, 2018 | UltIMMa-1/  NCT02684370 | Risankizumab (150 mg, at weeks 0, 4, and 16) | 304 | NA | 37 | NA |  | 102 | NA | 8 | NA |
| Gordon *et al*, 2018 | UltIMMa-2/  NCT02684357 | Risankizumab (150 mg, at weeks 0, 4, and 16) | 294 | NA | 21 | NA |  | 98 | NA | 4 | NA |
| Kristensen *et al*, 2022 | KEEPsAKE 1/  NCT03675308 | Risankizumab (150 mg, at weeks 0, 4, and 16) | 483 | 16 | 12 | NA |  | 481 | 14 | 20 | NA |
| Östör *et al*, 2022 | KEEPsAKE 2/  NCT03671148 | Risankizumab (150 mg, at weeks 0, 4, and 16) | 224 | 9 | 17 | NA |  | 219 | 8 | 12 | NA |
| Blauvelt *et al*, 2020 | NCT02672852 | Risankizumab (150 mg, at weeks 0, 4, and 16) | 407 | 21 | 6 | NA |  | 100 | 6 | 5 | NA |
| Reich *et al*, 2017 | reSURFACE 1/  NCT01722331 | Tildrakizumab (100 mg, at weeks 0, 4, and 16)  Tildrakizumab (200 mg, at weeks 0, 4, and 16) | 309  308 | 24  20 | 10  15 | NA |  | 154 | 8 | 9 | NA |
| Reich *et al*, 2017 | reSURFACE 2/  NCT01729754 | Tildrakizumab (100 mg, at weeks 0, 4, and 16)  Tildrakizumab (200 mg, at weeks 0, 4, and 16) | 307  314 | 41  35 | 0  0 | NA |  | 156 | 12 | 0 | NA |
| Papp *et al*, 2015 | NCT01225731 | Tildrakizumab (5 mg, at weeks 0, 4, and 16)  Tildrakizumab (25 mg, at weeks 0, 4, and 16)  Tildrakizumab (100 mg, at weeks 0, 4, and 16)  Tildrakizumab (200 mg, at weeks 0, 4, and 16) | 308 | 43 | 7 | NA |  | 45 | 9 | 0 | NA |

NA, not available; QW, every week; Q4W, every 4 weeks; Q8W, every 8 weeks; Q12W, every 12 weeks.

**Table S4.** Original Data of Outcomes Extracted from Included Open-label Extension Studies

| **Source** | **Trial Name/ ClinicalTrials.gov identifier** | **Treatment (dose)** | **Duration of Open-label Extension Period** | **EAIR of Serious Infection** | **EAIR of Infection** | **EAIR of NMSC** | **EAIR of Malignancies Excluding NMSC** | **EAIR of Nasopharyngitis** | **EAIR of Upper Respiratory Tract Infection** | **EAIR of Candida Infection** |
| --- | --- | --- | --- | --- | --- | --- | --- | --- | --- | --- |
| Papp *et al*, 2020 | AMAGINE-1/ NCT01708590 | Brodalumab (140mg or 210 mg, Q2W) | 1 year  120 weeks | NA  NA | NA  NA | NA  NA | NA  NA | NA  NA | NA  NA | 2.7  2.5 |
| Lebwohl *et al*, 2015 | AMAGINE-2/ NCT01708603 | Brodalumab (140mg or 210 mg, Q2W) | 52 weeks | 1.0 | NA | NA | 0.1 | NA | NA | 5.2 |
| Lebwohl *et al*, 2015 | AMAGINE-3/ NCT01708629 | Brodalumab (140mg or 210 mg, Q2W) | 52 weeks | 1.3 | NA | NA | 0.1 | NA | NA | 5.7 |
| Reich *et al*, 2022 | AMAGINE-2/ NCT01708603;  AMAGINE-3/ NCT01708629 | Brodalumab (140mg, Q2W)  Brodalumab (210 mg, Q2W) | 120 weeks | 0.6  1.5 | 84.2  105.9 | 0.6  0.5 | 0.2  0.6 | NA  NA | NA  NA | 3.2  4.5 |
| Lebwohl *et al*, 2019 | NCT01101100 | Brodalumab (210 mg, Q2W) | 1 year  2 years  3 years  4 years  5 years  6 years | 2.3  1.8  1.3  1.1  1.1  1.1 | NA  NA  NA  NA  NA  NA | NA  NA  NA  NA  NA  NA | NA  NA  NA  NA  NA  NA | NA  NA  NA  NA  NA  NA | NA  NA  NA  NA  NA  NA | 9.8  7.0  5.9  5.6  5.6  5.6 |
| Blauvelt *et al*, 2021 | UNCOVER-3/ NCT01646177 | Ixekizumab (160 mg at week 0, then 80 mg Q2W or Q4W) | 1 year  5 years | 0.8  1.0 | 52.6  17.6 | NA  NA | 0.6  0.6 | 18.0  7.5 | 7.3  2.8 | NA  1.7 |
| van der Heijde *et al*, 2018 | SPIRIT-P1/ NCT01695239 | Ixekizumab (160 mg at week 0, then 80 mg Q2W)  Ixekizumab (160 mg at week 0, then 80 mg Q4W) | 52 weeks | 1.5  3.0 | 50.4  50.5 | NA  NA | NA  NA | 12.0  13.4 | 7.5  10.4 | 3.8  1.5 |
| Chandran *et al*, 2020 | SPIRIT-P1/ NCT01695239 | Ixekizumab (160 mg at week 0, then 80 mg Q2W)  Ixekizumab (160 mg at week 0, then 80 mg Q4W) | 3 years | 0.7  1.8 | 24.8  24.5 | NA  NA | 0.5  0.7 | NA  NA | 5.0  6.9 | 1.1  1.4 |
| Genovese *et al*, 2018 | SPIRIT-P2/ NCT02349295 | Ixekizumab (160 mg at week 0, then 80 mg Q2W)  Ixekizumab (160 mg at week 0, then 80 mg Q4W) | 52 weeks | 1.3  2.3 | 60.4  61.6 | NA  NA | 0  1.1 | 11.6  12.5 | 20.6  10.3 | NA  NA |
| Gordon *et al*, 2014 | NCT01107457 | Ixekizumab (6 doses at 0, 2, 4, 8, 12, and 16 weeks, then 120 mg Q4W) | 52 weeks | 1.2 | NA | 0.6 | 0.6 | 7.1 | 5.3 | NA |
| Lacour *et al*, 2017 | JUNCTURE/ NCT01636687 | Secukinumab (150 mg or 300 mg, QW for 4 weeks, then Q4W) | 52 weeks | 3.1 | NA | NA | NA | 39.4 | 8.1 | 3.8 |
| Blauvelt *et al*, 2017 | CLEAR/  NCT02074982 | Secukinumab (300 mg, QW for 4 weeks, then Q4W) | 52 weeks | NA | 98.4 | NA | NA | 27.1 | 10.1 | NA |
| McInnes *et al*, 2017 | FUTURE2/ NCT01752634 | Secukinumab (75, 150 or 300 mg, QW for 4 weeks, then Q4W) | 104 weeks | 1.6 | 65.0 | 0.5 | 0.8 | 12.6 | 13.6 | 2.3 |
| Nash *et al*, 2018 | FUTURE3/ NCT01989468 | Secukinumab (150 or 300 mg, QW for 4 weeks, then Q4W) | 52 weeks | NA | NA | NA | NA | 20.2 | 10.1 | 3.2 |
| van der Heijde *et al*, 2020 | FUTURE5/ NCT02404350 | Secukinumab (150 mg, QW for 4 weeks, then Q4W)  Secukinumab (300 mg, QW for 4 weeks, then Q4W) | 52 weeks | 1.6  1.7 | NA  NA | NA  NA | 0.6  0.7 | 14.1  14.4 | 11.4  8.2 | 1.8  3.0 |
| Mease *et al*, 2021 | FUTURE5/ NCT02404350 | Secukinumab (150 or 300 mg, QW for 4 weeks, then Q4W) | 2 years | NA | NA | NA | NA | 9.4 | 7.5 | 0.6 |
| Bissonnette *et al*, 2018 | SCULPTURE/ NCT01640951 | Secukinumab (300mg, QW for 4 weeks, then Q4W, fixed interval) | 1 year  2 years  3 years  4 years  5 years | NA  NA  1.3  NA  NA | NA  NA  NA  NA  NA | NA  NA  NA  NA  NA | 0  0.6  0.4  0.3  0.4 | 20.1  17.2  14.1  16.1  15.0 | NA  NA  NA  NA  NA | 1.8  1.8  1.3  1.1  0.9 |
| Bissonnette *et al*, 2018 | SCULPTURE/ NCT01640951 | Secukinumab (300mg, QW for 4 weeks, then Q4W, retreated as needed) | 3 years | 1.7 | NA | NA | NA | 15.8 | NA | 0.9 |
| Coates *et al*, 2022 | BE ACTIVE/ NCT03347110 | Bimekizumab (160 mg Q4W); Bimekizumab (320 mg Q4W) | 152 weeks | 0.7 | NA | NA | 0.2 | 7.6 | 6.8 | 4.6 |
| Strober *et al*, 2023 | BE RADIANT/  NCT03536884 | Bimekizumab (320 mg Q4W) | 1 year  2 years | 2.4  0.8 | NA  NA | 0.9  0.3 | NA  NA | 30.0  11.8 | 13.1  3.9 | 26.9  11.2 |
| Reich *et al*, 2020 | VOYAGE 1/ NCT02207231; VOYAGE 2/ NCT02207244 | Guselkumab (100 mg, at weeks 0 and 4, then Q8W) | 100 weeks  156 weeks | 1.06  1.15 | 81.74  74.03 | 0.39  0.28 | 0.38  0.47 | NA  NA | NA  NA | NA  NA |
| Blauvelt *et al*, 2022 | VOYAGE 1/ NCT02207231; VOYAGE 2/ NCT02207244 | Guselkumab (100 mg, at weeks 0 and 4, then Q8W) | 5 years | 0.85 | NA | 0.34 | 0.45 | NA | NA | NA |
| Coates *et al*, 2022 | COSMOS/  NCT03796858 | Guselkumab (100 mg, at weeks 0 and 4, then Q8W) | 56 weeks | 0.8 | 37.2 | NA | NA | 6.3 | 3.9 | NA |
| McInnes *et al*, 2020 | DISCOVER-2/  NCT03158285 | Guselkumab (100 mg, Q4W)  Guselkumab (100 mg, at weeks 0 and 4, then Q8W) | 52 weeks | 1.26  1.24 | 34.09  34.89 | NA  NA | 0  0.83 | 9.0  NA | 10.0  NA | NA  NA |
| McInnes *et al*, 2022 | DISCOVER-2/  NCT03158285 | Guselkumab (100 mg, Q4W)  Guselkumab (100 mg, at weeks 0 and 4, then Q8W) | 112 weeks | 1.0  2.2 | 35.8  40.5 | NA  NA | NA  0.2 | NA  NA | NA  NA | NA  NA |
| Gooderham *et al*, 2022 | UltIMMa-1/ NCT02684370;  UltIMMa-2/ NCT02684357 | Risankizumab (150 mg, at weeks 0 and 4, then Q12W) | 52 weeks | 1.8 | NA | 0.5 | 0 | NA | NA | NA |
| Gooderham *et al*, 2022 | LIMMitless/ NCT03047395 | Risankizumab (150 mg, at weeks 0 and 4, then Q12W) | 172 weeks | 1.1 | NA | 0.6 | 0.4 | NA | NA | NA |
| Papp *et al*, 2021 | LIMMitless/ NCT03047395 | Risankizumab (150 mg, at weeks 0 and 4, then Q12W) | 208 weeks | 1.2 | NA | 0.5 | 0.3 | 17.3 | 10.7 | NA |
| Reich *et al*, 2020 | reSURFACE 1/  NCT01722331;  reSURFACE 2/  NCT01729754 | Tildrakizumab (100 mg, at weeks 0 and 4, then Q12W)  Tildrakizumab (200 mg, at weeks 0 and 4, then Q12W) | 148 weeks | 1.14  1.12 | NA  NA | 0.50  0.49 | 0.55  0.39 | 10.18  9.82 | 3.77  5.57 | NA  NA |
| Thaci *et al*, 2021 | reSURFACE 1/  NCT01722331;  reSURFACE 2/  NCT01729754 | Tildrakizumab (100 mg, at weeks 0 and 4, then Q12W)  Tildrakizumab (200 mg, at weeks 0 and 4, then Q12W) | 256 weeks | 1.2  1.3 | NA  NA | 0.4  0.4 | 0.7  0.6 | 10.5  10.7 | 3.8  4.6 | NA  NA |

The data are presented as the number of events per 100 PYs. PY, patient‐years.

EAIR, exposure-adjusted incidence rate; NMSC, nonmelanoma skin cancer; NA, not available; QW, every week; Q4W, every 4 weeks; Q8W, every 8 weeks; Q12W, every 12 weeks.

# 2 Supplementary Figures

**Supplementary Figure 1.** Evaluation of risk of bias of included publications regarding (A) IL-17 inhibitors, and (B) IL-23 inhibitors.

**Supplementary Figure 2.** Subgroup analysis by indication of risk ratio (RR) of serious infection with the treatment of IL-17 inhibitors vs placebo.

**Supplementary Figure 3.** Subgroup analysis by indication of risk ratio (RR) of overall infection with the treatment of IL-17 inhibitors vs placebo.

**Supplementary Figure 4.** Subgroup analysis by indication of risk ratio (RR) of malignancy with the treatment of IL-17 inhibitors vs placebo.

**Supplementary Figure 5.** Pooled risk ratio (RR) of nasopharyngitis with the treatment of IL-17 inhibitors vs placebo. (A) Subgroup analysis by biologic; (B) Subgroup analysis by indication.

**Supplementary Figure 6.** Pooled risk ratio (RR) of upper respiratory tract infection with the treatment of IL-17 inhibitors vs placebo. (A) Subgroup analysis by biologic; (B) Subgroup analysis by indication.

**Supplementary Figure 7.** Pooled risk ratio (RR) of Candida infection with the treatment of IL-17 inhibitors vs placebo. (A) Subgroup analysis by biologic; (B) Subgroup analysis by indication.

**Supplementary Figure 8.** Subgroup analysis by indication of risk ratio (RR) of serious infection with the treatment of IL-23 inhibitors vs placebo.

**Supplementary Figure 9.** Subgroup analysis by indication of risk ratio (RR) of overall infection with the treatment of IL-23 inhibitors vs placebo.

**Supplementary Figure 10.** Subgroup analysis by indication of risk ratio (RR) of malignancy with the treatment of IL-23 inhibitors vs placebo.

**Supplementary Figure 11.** Pooled risk ratio (RR) of nasopharyngitis with the treatment of IL-23 inhibitors vs placebo. (A) Subgroup analysis by biologic; (B) Subgroup analysis by indication.

**Supplementary Figure 12.** Pooled risk ratio (RR) of upper respiratory tract infection with the treatment of IL-23 inhibitors vs placebo. (A) Subgroup analysis by biologic; (B) Subgroup analysis by indication.

**Supplementary Figure 13.** Funnel plots of outcomes of (A) serious infection; (B) overall infection; (C) malignancy; (D) nasopharyngitis; (E) upper respiratory tract infection; (F) Candida infection with the treatment of IL-17 inhibitors.

**Supplementary Figure 14.** Funnel plots of outcomes of (A) serious infection; (B) overall infection; (C) malignancy; (D) nasopharyngitis; (E) upper respiratory tract infection with the treatment of IL-23 inhibitors.

**Supplementary Figure 15.** Sensitivity analyses of outcomes of (A) serious infection; (B) overall infection; (C) malignancy with the treatment of IL-17 inhibitors.

**Supplementary Figure 16.** Sensitivity analyses of outcomes of (A) nasopharyngitis; (B) upper respiratory tract infection; (C) Candida infection with the treatment of IL-17 inhibitors.

**Supplementary Figure 17.** Sensitivity analyses of outcomes of (A) serious infection; (B) overall infection; (C) malignancy with the treatment of IL-23 inhibitors.

**Supplementary Figure 18.** Sensitivity analyses of outcomes of (A) nasopharyngitis; (B) upper respiratory tract infection with the treatment of IL-23 inhibitors.

# 3 Supplementary Information

**Detailed searching strategy and results**

- **Pubmed:**

(("Psoriasis"[Mesh]) OR ("Arthritis, Psoriatic"[Mesh])) AND (("Interleukin-17"[Mesh]) OR ("Interleukin-23"[Mesh]))

Final query: 1824

- **Medline:**

Ovid MEDLINE(R) ALL <1946 to May 17, 2023>

1 exp Psoriasis/ 48630

2 exp Arthritis, Psoriatic/ 8281

3 exp Interleukin-17/ai [Antagonists & Inhibitors] 963

4 exp Interleukin-23/ai [Antagonists & Inhibitors] 441

5 1 or 2 48631

6 3 or 4 1290

7 5 and 6 557

- **Web of Science:**

(TS=(psoriasis) OR TS=(psoriatic arthritis)) AND (TS=(IL-17 inhibitor*) OR TS=(IL-23 inhibitor*))

Final query: 1831

- **ClinicalTrials.gov:**

Psoriasis OR Psoriatic arthritis (condition/disease) + Brodalumab (intervention/treatment) + filter (with results): 12

Psoriasis OR Psoriatic arthritis (condition/disease) + Ixekizumab (intervention/treatment) + filter (with results): 23

Psoriasis OR Psoriatic arthritis (condition/disease) + Secukinumab (intervention/treatment) + filter (with results): 63

Psoriasis OR Psoriatic arthritis (condition/disease) + Bimekizumab (intervention/treatment) + filter (with results): 12

Psoriasis OR Psoriatic arthritis (condition/disease) + Guselkumab (intervention/treatment) + filter (with results): 14

Psoriasis OR Psoriatic arthritis (condition/disease) + Risankizumab (intervention/treatment) + filter (with results): 17

Psoriasis OR Psoriatic arthritis (condition/disease) + Tildrakizumab (intervention/treatment) + filter (with results): 6

Final query: 147
